# Development of 5‐Amino‐2,4,6‐triiodoisophthalic Acid Derivatives for Carbamoylation of Amino Acids

**DOI:** 10.1002/open.202500174

**Published:** 2025-09-12

**Authors:** Kousaku Ohkawa, Tracy Nguyen, Chloe Jin, Hemdeep Kaur, Beatrice Mae Malvar, Rebecca Back, Parisa Khosropour, Shuichi Suzuki, Frank P. K. Hsu, Ichiro Yuki

**Affiliations:** ^1^ Division of Synthetic Polymers Institute of High Polymer Research Faculty of Textile Science and Technology Shinshu University Tokida 3‐15‐1 Ueda, Nagano Prefecture 386–8567 Japan; ^2^ Division of Bibliometrics and Social Implementation Institute for Fiber Engineering Interdisciplinary Cluster for Cutting Edge Research Shinshu University Tokida 3‐15‐1 Ueda, Nagano Prefecture 386–8567 Japan; ^3^ Department of Neurological Surgery University of California, Irvine 200S Manchester St., Suite 210 Orange CA 92868 USA; ^4^ AquaTeX Medical, Inc. Irvine CA 92617 USA; ^5^ Department of Neurosurgery The Jikei University Hospital 3 Chome‐25−8 Nishi‐Shinbashi Minato City, Tokyo 105–8461 Japan

**Keywords:** carbamoylation, di(acetoxyethyl) 5‐amino‐2,4,6‐triiodoisophthalate, diethyl 5‐amino‐2,4,6‐triiodoisophthalate, radiopaque material, selective deprotection

## Abstract

Herein, 5‐amino‐2,4,6‐triiodoisophthalic acid (ATIIPA) is used as a nucleophile to produce the corresponding 1,3‐diesters. Two types of 1,3‐diesters, i) diethyl 5‐amino‐2,4,6‐triiodoisophthalate (DEtTIIP) and ii) diacetoxyethyl 5‐amino‐2,4,6‐triiodoisophthalate (DAcOEtTIIP), are mostly prepared in quantitative yields. The 1,3‐esters are tested as a carbamoylation agent toward the amino groups of the *β*Ala esters via isocyanation of the 5‐amino group. The addition reaction of DEtTIIP‐NCO and *β*Ala‐OEt yields DEtTIIP:CO‐*β*Ala‐OEt, and the 1,3‐diethyl ester is highly resistant to alkaline hydrolysis due to the steric shielding by the adjacent 2,4,6‐iodines, while the *α*‐ethyl ester of the *β*Ala substructure is easily removed. Alkaline hydrolysis of another adduct, DAcOEtTIIP:CO‐*β*Ala‐O^t^Bu, removes only the 1,3‐acetoxy ethyl groups to form the product DAcOHTIIP:CO‐*β*Ala‐O^t^Bu, and the acidic fission of the ‐O^t^Bu ester is quantitative to give DAcOEtTIIP:CO‐*β*Ala. These results indicate that the DAcOEtTIIP is a feasible precursor for the *N*‐carbamoylation of the amino acid esters, preserving the freedom for selective ester deprotection, which further inspires the design of contrast molecules using amino acids and peptides.

## Graphical abstract

The *N*‐carbamolyation agents described in this study are prepared in quantitative yields and adaptable for selective conversion of esters to carboxylic acids, which are utilized for the synthesis of radiopaque compounds/peptides or polymeric materials: * denotes the reactive isocyanate carbon, sterically hindered carbonyls inactive to nucleophiles; #, carbonyls susceptible to alkaline hydrolysis after carbamylation.

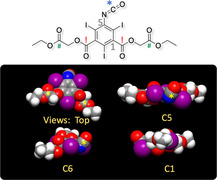

## 1 Introduction

5‐Amino‐2,4,6‐triiodoisophthalic acid (ATIIPA) is one of the major precursors for designing the iodine‐based radiopaque, which is used with X‐ray imaging or computed tomography instruments.^[^ 1, 2, 3 ^]^ Iohexol and iopamidol are examples of the monomeric derivatives of ATIIPA, and both have multiple dihydroxy propyl groups that provide a solubility in water. The iodine‐based contrast agent products comprised of the monomeric ATIIPA derivatives are hyperosmotic as high as ≈800 mM at the iodine content [I] of 300 mg mL^–^^1^, corresponding to fourfold to fivefold of the body fluid osmolality, which is a risk that causes adverse complications.^[^ ^4^, ^5^ ^–^ ^6^ ^]^ Multimerization of the ATTIPA substructures is one of the approaches for reducing the hyperosmolality of the iodine‐based contrast agent products, for instance, as seen in the chemical structure of Iodixanol,^[^ ^7^ ^,^ ^8^ ^]^ a dimer of the ATIIPA substructures, or the higher multimers.^[^ ^9^ ^,^ ^10^ ^]^ Establishment of the synthetic routes for the ATIIPA derivative requires specific considerations regarding the steric effects brought by the 2,4,6‐tiiode substituents.

**Figure 1:**
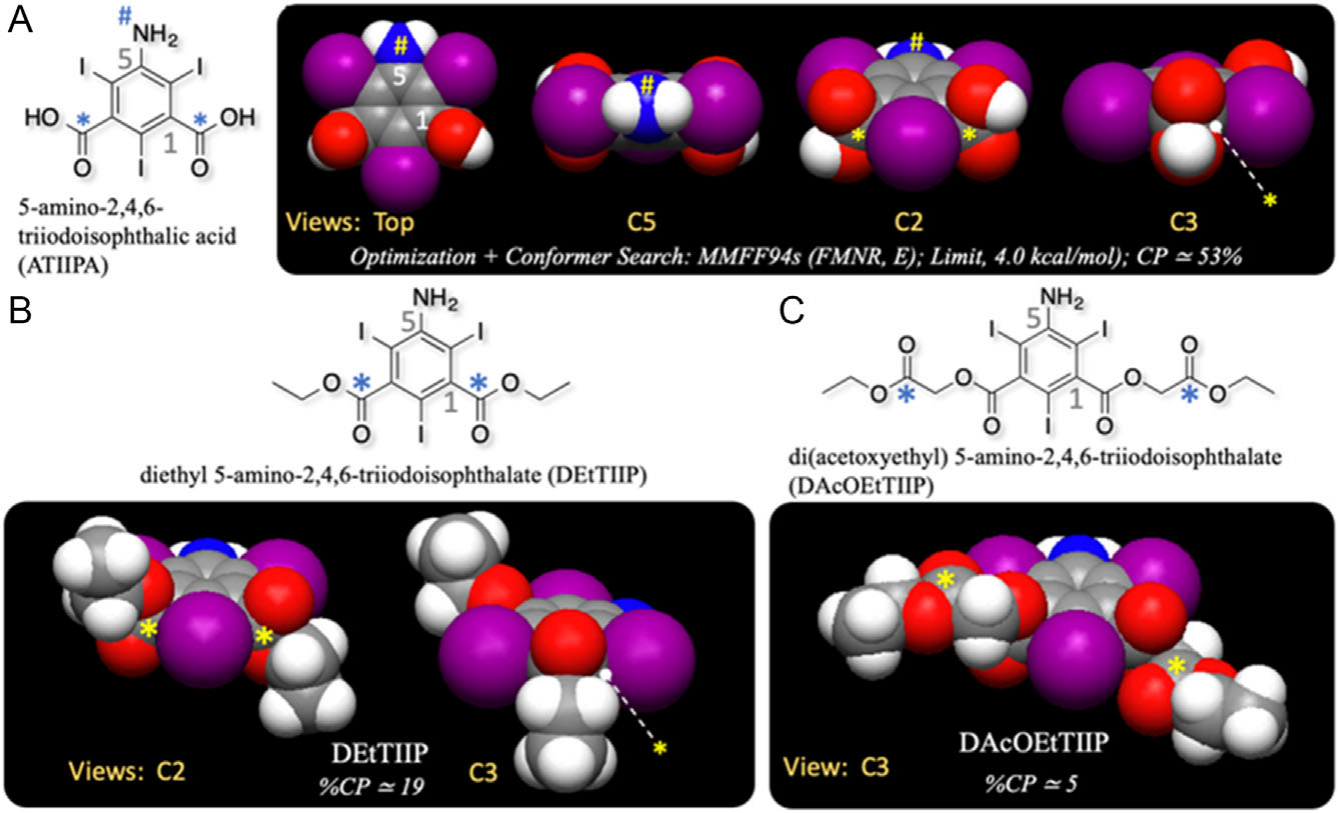
Top conformations from conformer search calculations of A) ATIIPA, B) DEtTIIP, and C) DAcOEtTIIP, with multiple views of the aromatic carbons. Asterisks (*) in (A) and (B) denote the iodine‐shielded carbonyl carbons, while those in (C) are the ones accessible by the nucleophiles.

In a previous study, the authors hypothesized the iodine shielding effect for 3,5‐bis(acetamido)‐2,4,6‐triiodobenzoic acid, also known as diatrizoic acid (DTZOH), where the 1‐carboxyl carbon is blocked by the adjacent, bulky iodine elements.^[^ ^11^ ^]^ As a result, DTZOH is not active as an acylating agent, whereas the 1‐carboxyl oxygens are active as nucleophiles and easily undergo esterification using alkyl halides. The chemical structure of ATIIPA is a derivative of isophthalic acid having a 2,4,6‐triiodophenyl substructure, and the 1,3‐carboxylic acid carbons are affected by the iodine‐shielding. **Figure** **1**A represents the results of a conformer search calculation for ATIIPA, and as expected, the 1,3‐carboxylic groups have almost perpendicular dihedrals to the phenyl ring. The C2 and C3 views of Figure 1A suggest that the 1,3‐carboxylic carbons are not active enough to be attacked by the foreign nucleophiles, but the carboxylic oxygens of ATIIPA are assumed to be active as the nucleophiles.

One of the ATIIPA derivatives, diethyl 5‐amino‐2,4,6‐triiodoisophthalate (DEtTIIP), was originally reported by a preparation method using 5‐amino‐2,4,6‐triiodoisophthaloyl dichloride, a corresponding acid halide of ATIIPA, and an excess amount of ethanol at refluxing temperature for 4 h.^[^ ^12^ ^]^ However, in this report, the molar yield of DEtTIIP was not described, and the product was used for crystallographic analysis. To evaluate the assumption described above, the authors in this study first performed the base‐catalyzed esterification of ATIIPA using five molar excesses of bromoethane (Figure S1A, Supporting Information) and NaHCO_3_ in DMF at 40 °C for 18 h. The yield was mostly quantitative (98 mol%) and the product was identified as DEtTIIP by infrared (Figure S1, Supporting Information), ^1^H‐ (Figure S2A, Supporting Information), and ^13^C‐CPD‐NMR (Figure S2B, Supporting Information) spectroscopies. It was also important to note that no sign was found to indicate *N* ^5^‐ethylation during the esterification reaction, suggesting that the 5‐amino group of ATIIPA is not reactive under the mild conditions described in the Supporting Information.

Figure 1B represents the top‐ranked conformations from the conformer search calculations of DEtTIIP. The 1,3‐cabonyl carbons of DEtTIIP also have mostly perpendicular dihedral angles toward the aromatic ring, and DEtTIIP is recognized as a 1,3‐carboxy‐protected derivative of ATIIPA. The 1,3‐diethyl ester carbonyl carbons (marked as * in Figure 1B) are, on the other hand, sterically hindered by the adjacent 2,4,6‐iodines, and hence, the nucleophilic approaches toward the DEtTIIP 1,3‐carbonyls are strongly restricted, leading to a hypothesis that DEtTIIP is presumed highly resistant to alkaline hydrolysis to regenerate ATIIPA. The successful yield of DEtTIIP (Figure S1–S3, Supporting Information) inspires another derivatized molecule of ATIIPA, 1,3‐di(acetoxyethyl) 5‐amino‐2,4,6‐triiodoisophthalate, which is abbreviated as DAcOEtTIIP and has so‐called ethyl‐protected esters (acetoxyethyl groups) on the 1,3‐carboxylates of ATIIPA.

Figure 1C suggests that the 1,3‐acetoxyethyl carbonyl carbons (Figure 1C, marked with *) of DAcOEtTIIP are not affected by the iodine‐shielding, so that these carbonyls are expected to be susceptible to alkaline hydrolysis, whereas the carbonyl carbons of the 1,3‐carboxylates are not. This study was conducted to acquire experimental evidence for the above differences in the two types of carbonyl carbons, with or without iodine shielding. The 5‐amino groups of DEtTIIP and DAcOEtTIIP are surrounded by adjacent 2,6‐iodines, and the nucleophilic reactivities of these types of amino groups are described in patents^[^ ^9^ ^,^ ^13^ ^]^ and original articles,^[^ ^14^ ^,^ ^15^ ^]^ in order to convert the phenyl amine, surrounded by bulky adjacent substituents, to the corresponding isocyanate. This study also investigated DEtTIIP and DAcOEtTIIP as the carbamoylation agents, particularly toward amino acid derivatives.

## 2 Results and Discussion

### 2.1 Base‐Catalyzed Esterification of ATIIPA

In our previous study,^[^ ^11^ ^]^ the base‐catalyzed esterification of diatrizoic acid (DTZOH) well proceeded using 2–3 eq.mol of tert‐butyl *α*‐bromoacetate and NaHCO_3_ in DMF in a quantitative manner. Since the 1‐carboxylic carbon of the DTZOH is sterically shielded by two vicinal iodine elements, the carbonyl is resistant to the nucleophile approach, so that DTZOH is not appropriate for use as an acylating agent. The carboxylic oxygen, on the other hand, is not affected by such a hindrance and can be used as a nucleophile in the esterification reaction. The 1,3‐dicarboxylic acids in ATIIPA have similar steric conditions found in DTZOH, and hence, the 1,3‐carboxylic oxygens of ATIIPA would work as nucleophiles.

**Figure 2:**
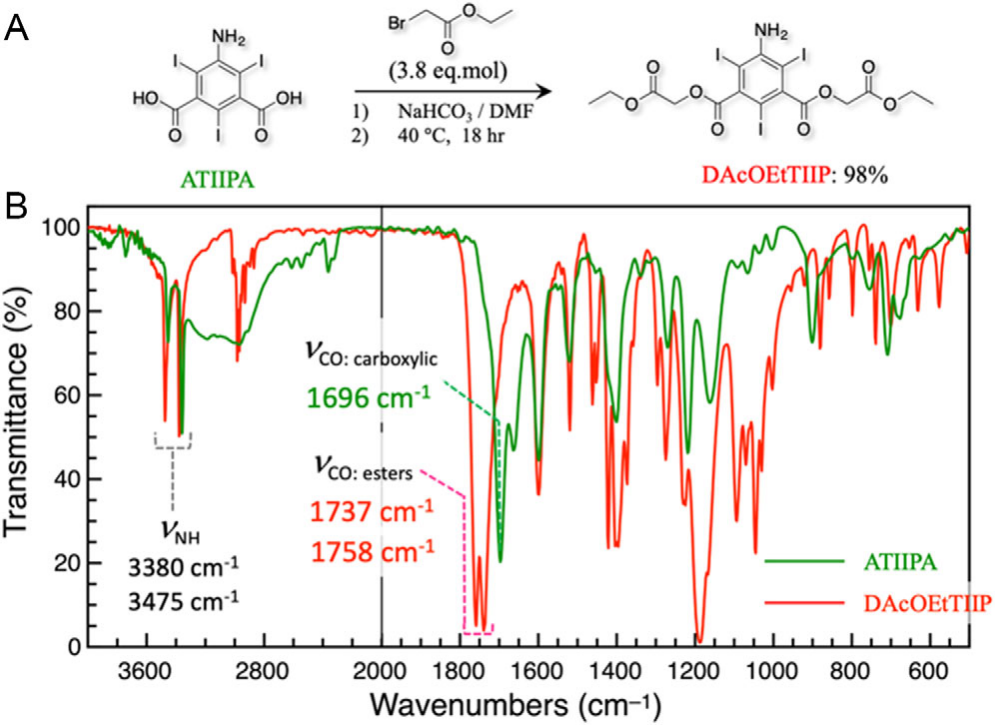
A) Base‐catalyzed esterification of ATIIPA using ethyl *α*‐bromoacetate to produce DAcOEtTIIP, and B) infrared spectra of the ATIIPA substrate and the DAcOEtTIIP product.

**Figure** **2**A represents the base‐catalyzed preparation of DAcOEtTIIP with a reaction yield of 98 mol%, suggesting that the 1,3‐carboxylic oxygen with the vicinal, bulky iodine elements is still active as the nucleophile. The FT‐IR spectra of DAcOEtTIIP (Figure 2B) indicated two types of C=O stretching vibrations of the esters (1737 and 1758 cm^−1^), which were shifted from the carboxylic C=O stretching vibration of the parent ATIIPA found at 1696 cm^−1^, while retaining the two modes of the NH stretching vibrations of the 5‐amino group (3380 and 3475 cm^−1^).

**Figure 3:**
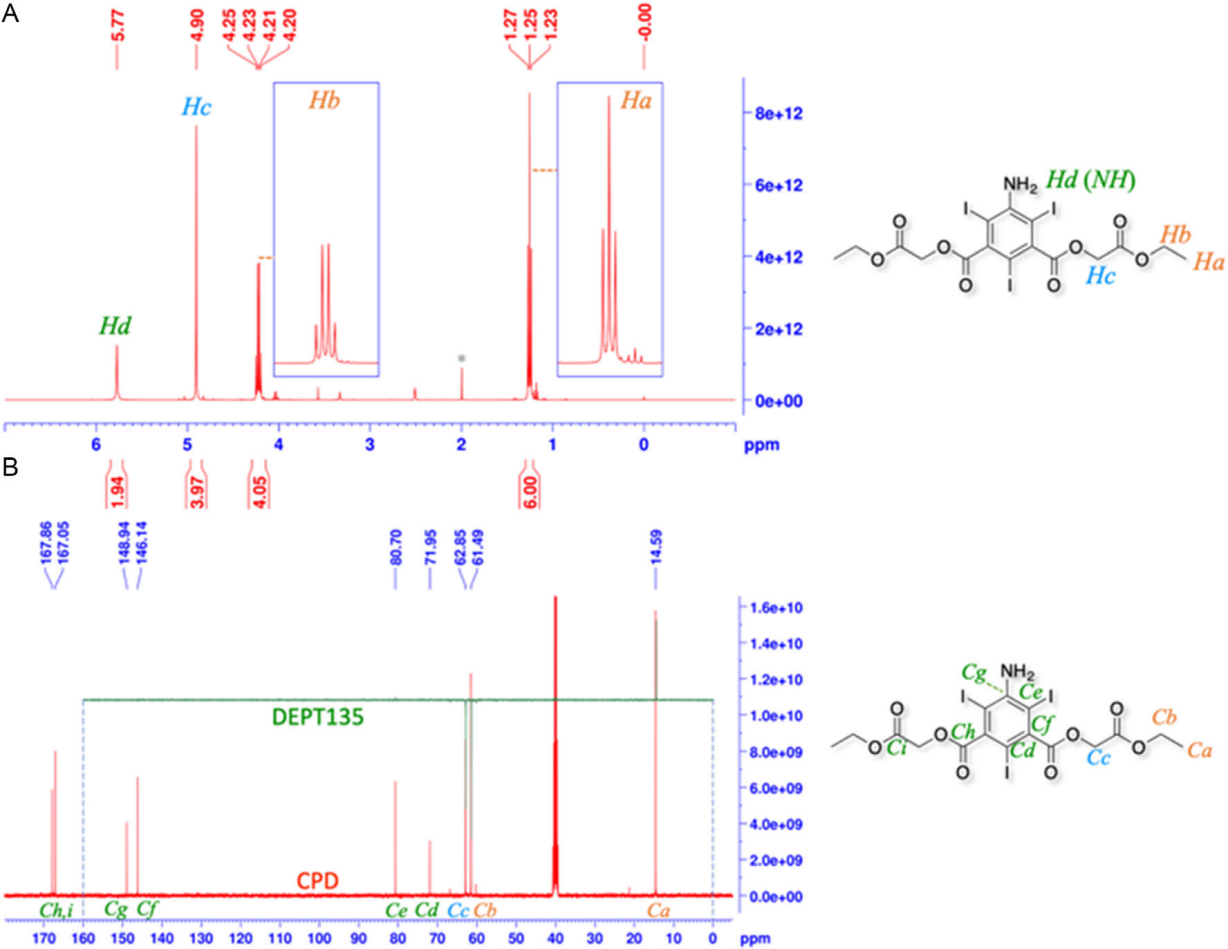
A) ^1^H‐ and B) ^13^C‐CPD/DEPT135 spectra of DAcOEtTIIP.

**Figure** **3**A represents the ^1^H‐NMR spectrum (DMSO*d*6, TMS) of DAcOEtTIIP. The protons on the methyl (*H*_*a*_, *δ* (ppm) = 1.25, t, 6H) and methylene (*H*_*b*_, 4.22, q, 4H) groups comprising the ethyl esters bound to the acetoxy moiety of DAcOEtTIIP were clearly observed with the expected integrals. Four protons on the acetoxymethylene moiety (*H*_*c*_, 4.90, s, 4H) were also found, as well as the 5‐amino protons (*H*_*d*_, 5.77, s, 2H). In the ^13^C‐CPD/DEPT135 NMR (Figure 3B, DMSO*d*6, TMS), the carbons of the methyl (*C*_*a*_, *δ* (ppm) = 14.6, upward) and methylene (*C*_*b*_, 61.5, downward) groups of the ethyl ester were observed, along with the acetoxymethylene (*C*_*c*_, 62.9, downward) carbons. The four aromatic carbons of DAcOEtTIIP were assigned to C^2^ (*C*_*d*_, 72.0), C^4,6^ (*C*_*e*_, 80.7), C^1,3^ (*C*_*f*_, 146.1), and C^5^ (*C*_*g*_, 148.9). The 1,3‐carbonyl carbons of the isophthalate (*C*_*h*_, 167.1) and the 1,3‐acetoxy carbonyl groups (*C*_*i*_, 167.9) were assigned to identify the structure of the DAcOEtTIIP.

### 2.2 Isocyanation of DEtTIIP and DAcOEtTIIP Using Bis(trichloromethyl) Carbonate

Bis(trichloromethyl) carbonate (TCMC) is a conventional chemical to generate COCl_2_ in situ, and the use of TCMC with a small amount of dried charcoal powder ensured the almost quantitative decomposition of TCMC to 3.0 eq.mol of COCl_2_ upon mild heating, which we have already applied to the synthesis of the amino acid *N*‐carboxylic anhydrides.^[^ ^16^ ^,^ ^17^ ^]^ The same method was performed to convert DEtTIIP and DAcOEtTIIP to the corresponding isocyanates upon mild heating. DEtTIIP was converted to the corresponding isocyanate using 0.5 eq.mol of TCMC (1.5 eq.mol of COCl_2_ in situ) in mostly quantitative yield (Figure S3A, Supporting Information), and the stretching vibration of —N=C=O was found at 2238 cm^−1^, while retaining the stretching vibrations of the 1,3‐diethyl esters.

**Figure 4:**
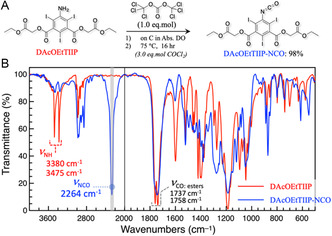
A) Conversion of 5‐amino to 5‐isocyanato groups of DAcOEtTIIP to give DAcOEtTIIP‐NCO and B) infrared spectra of the DAcOEtTIIP substrate and the DAcOEtTIIP‐NCO product.

Similarly, **Figure** **4** represents the isocyanation reaction of DAcOEtTIIP and the FT‐IR spectra of the substrate and the product. After the reaction using 1.0 eq.mol TCMC (3.0 eq.mol COCl_2_ in situ) at 75 °C for 16 h, an oily product was recovered in 98 mol% yield. The FT‐IR spectrum of the product (Figure 4B, blue trace) showed a remarkable vibrational absorption at 2264 cm^−1^, which was assigned to the stretching vibration of the —N=C=O group, and two modes of the N—H stretching vibrations found in the DAcOEtTIIP substrate (3380 and 3475 cm^−1^) were almost completely decayed in the product spectrum. The coupling vibrations of the two ester C=O groups were retained after the conversion. The spectral observations indicated that the DAcOEtTIIP substrate was converted to the corresponding isocyanate, 1,3‐di(acetoxyethyl) 5‐isocyanato‐2,4,6‐triiodoiso‐phthalate (DAcOEtTIIP‐NCO).

### 2.3 N‐Carbamoylation of Amino Acid Ester Using DEtTIIP‐NCO

**Figure 5:**
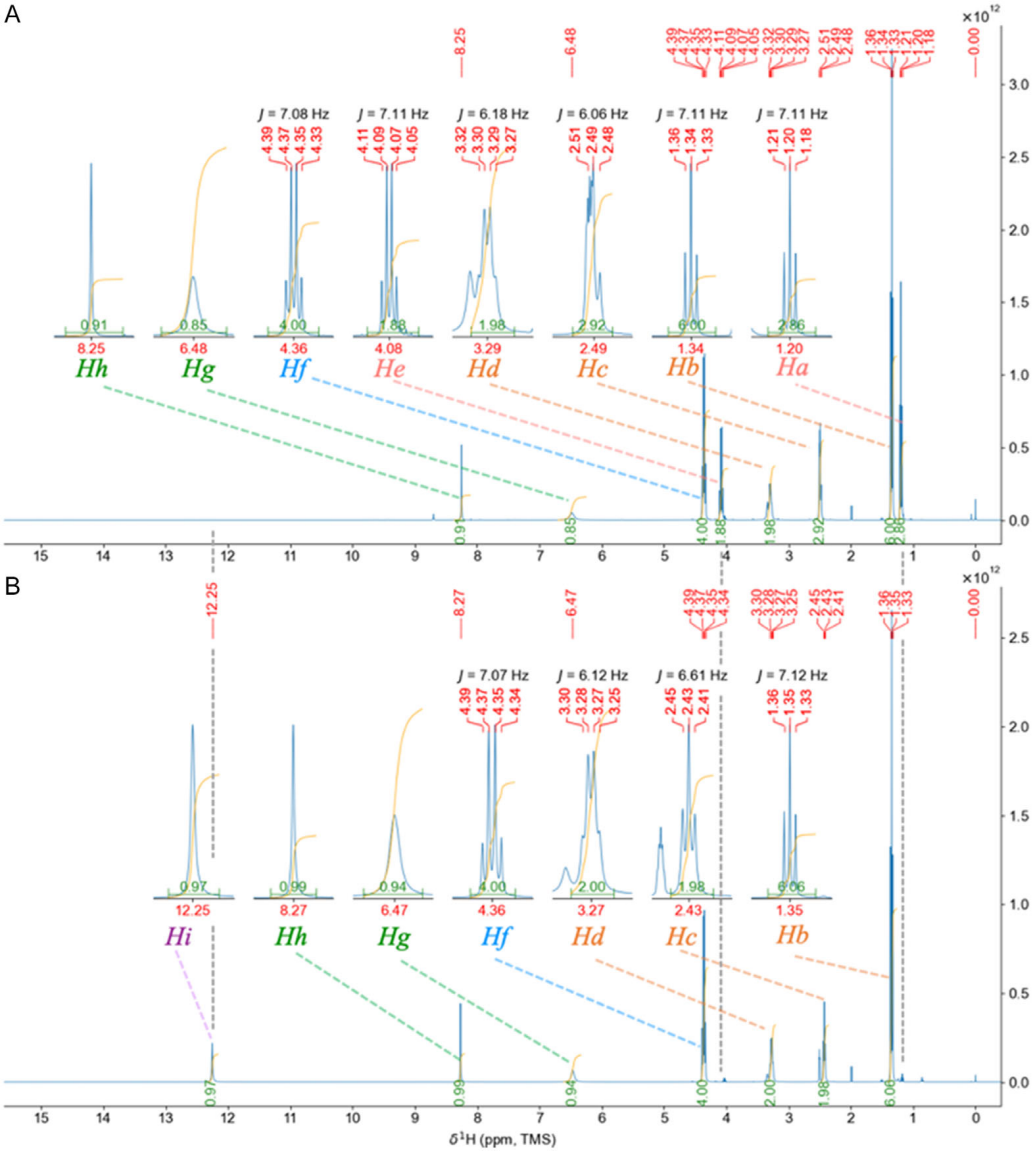
Comparison of ^1^H‐NMR spectra before and after the 5.0 eq.mol alkaline treatment of A) the DEtTIIP:CO‐*β*Ala‐OEt substrate and B) the DEtTIIP:CO‐*β*Ala product.

The *β*‐alanine ethyl ester was chosen as the substrate for the *N* ^*β*^‐carbamoylation using DEtTIIP‐NCO, and the addition reaction of the substrate at 70 °C for 18 h gave the product, diethyl 5‐(carbonyl‐*β*‐alanyloxyethyl)amino‐2,4,6‐triiodoisophthal‐ate (DEtTIIP:CO‐*β*Ala‐OEt), in quantitative yield. The ^1^H‐NMR spectrum of the product (**Figure** **5**A) included the proton signals on the methyl (*H*_*a*_, *δ* (ppm) = 1.20, t, 3H) and the methylene (*H*_*e*_, 4.08, q, 2H) groups of the ethyl ester on the *β*Ala moiety. The proton signals of the 1,3‐diethyl esters on isophthalate were assigned to the methyl (*H*_*b*_, 1.34, t, 6H) and the methylene (*H*_*f*_, 4.36, q, 4 H) groups. The proton signals on C^*α*^ and C^*β*^ of the *β*Ala substructure were found as (*H*_*c*_, 2.49, m, 2.9H, overlapped with the solvent signal) and (*H*_*d*_, 3.29, q, 2H), respectively. Signals on the urea substructure as 5‐aminocarbonyl (*H*_*h*_, 8.27, s, 1H) and *β*‐aminocarbonyl (*H*_*i*_, 12.3, s 1H) were detected.

**Figure 6:**
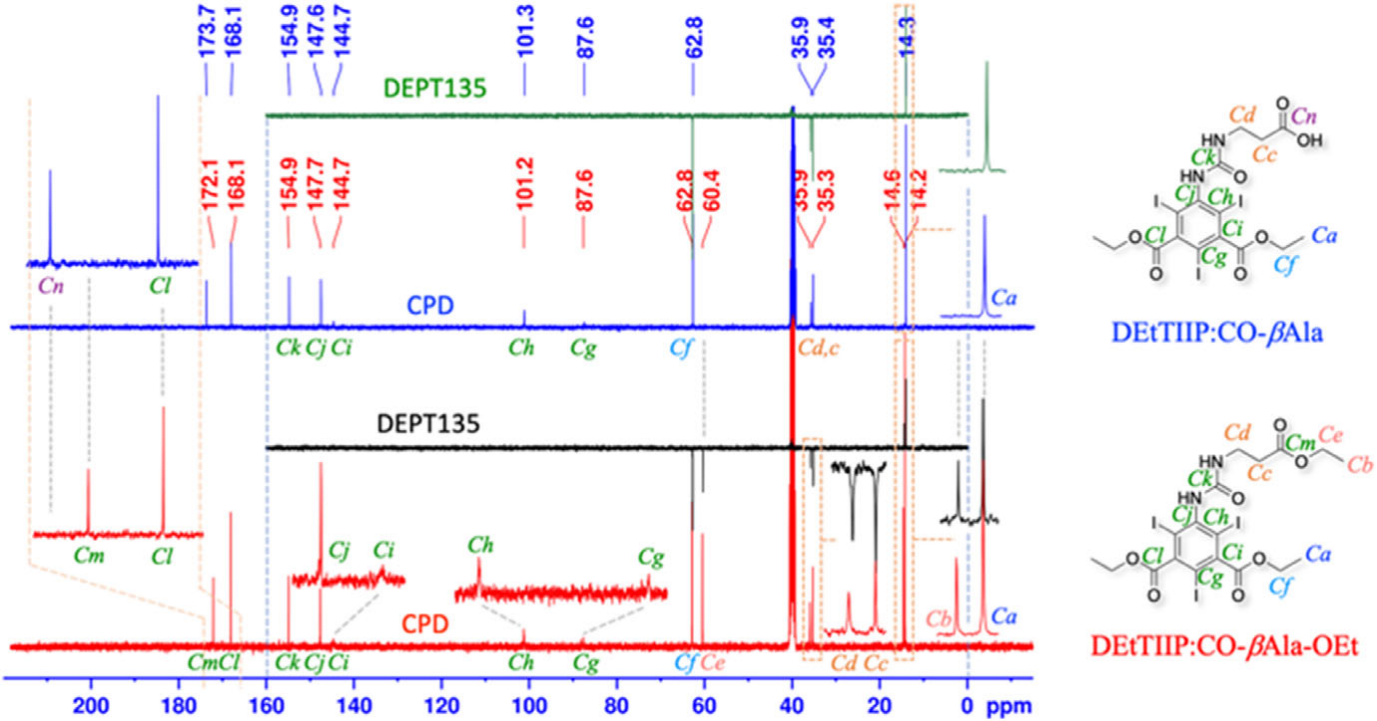
Comparison of ^13^C‐CPD/DEPT135‐NMR spectra before and after the excess alkaline treatment of the DEtTIIP:CO‐*β*Ala‐OEt substrate (red and black traces) and the DEtTIIP:CO‐*β*Ala product (blue and green traces).

In the ^13^C‐CPD/DEPT135 spectra of DEtTIIP:CO‐*β*Ala‐OEt (**Figure** **6**, red and black traces), the signals of the *β*Ala ethyl ester were assigned to the methyl (*C*_*a*_, *δ* (ppm = 14.6), upward) and the methylene (*C*_*e*_, 60.4, downward) carbons as well as those of the 1,3‐di(ethyl ester) on the isophthalate substructure were assigned to the methyl (*C*_*b*_, 14.2, upward) and the methylene (*C*_*f*_, 62.8) carbons. The C^*α*^ and C^*β*^ carbons of the *β*Ala moiety were detected as (*C*_*c*_, 35.4, downward) and (*C*_*d*_, 35.9, downward), respectively. The four aromatic carbons on the DEtTIIP substructure were assigned as C^2^ (*C*_*g*_, 87.6), C^4,6^ (*C*_*h*_, 101.3), C^1,3^ (*C*_*i*_, 144.7), and C^5^ (*C*_*j*_, 147.7). Three signals of the carbonyl carbons were the substructures of the urea carbonyl (*C*_*k*_, 154.9), the 1,3‐carbonyls (*C*_*l*_, 168.1) of the isophthalate, and the *β*Ala carbonyl (*C*_*m*_, 172.1).

**Figure 7:**
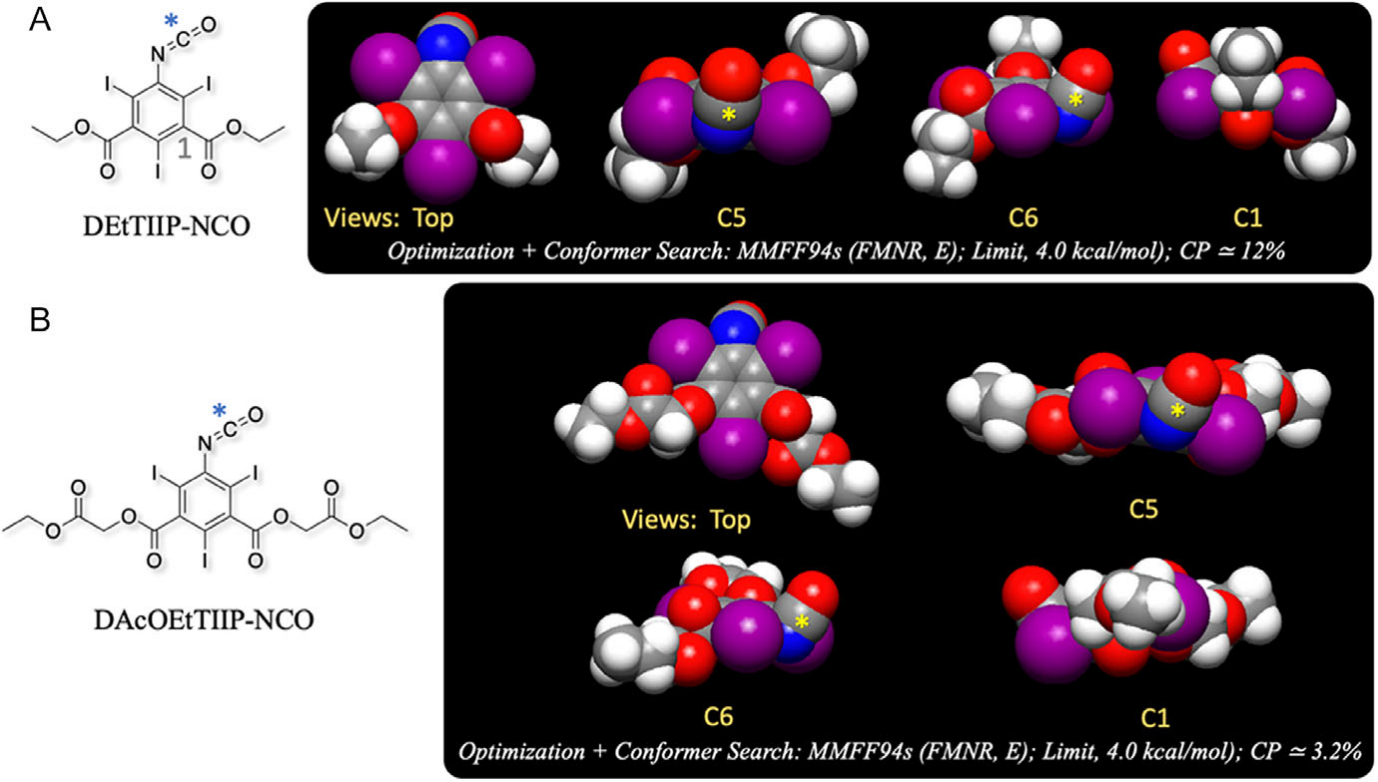
Top conformations from conformer search calculations of A) DEtTIIP‐NCO and B) DAcOEtTIIP‐NCO with multiple views from the aromatic carbons. Asterisks (*) in (A) and (B) denote the 5‐isocyanato carbons, which are attacked by the nucleophiles.

**Figure** **7**A represents the rank 1 conformation of DEtTIIP from the conformer search calculation. The C5 and C6 views suggest that the nucleophilic center carbon (*) of the 5‐isocyanato group on DEtTIIP is mostly free from iodine‐shielding, so that the nucleophiles, that is, the *β*‐amino groups of *β*Ala, can approach the center for the addition reaction.

The results of the NMR spectroscopies and the conformer calculation indicated that the production of DEtTIIP:CO‐*β*Ala‐OEt via the addition reaction between the 5‐isocyanato and the *β*‐amino groups are possible to occur and produce the expected product.

### 2.4 Resistance of the 1,3‐Diethyl Esters in DEtTIIP against Alkaline Hydrolysis

The 1,3‐carbonyl carbons of DEtTIIP are sterically hindered by the adjacent 2,4,6‐iodines (Figure 1B and 7A), and hence, the carbonyls are assumed to be inactive for nucleophilic attack. The inactivity of the 1,3‐carbonyl carbons was tested by alkaline hydrolysis using an aqueous NaOH solution, in which the DEtTIIP:CO‐*β*Ala‐OEt substrate was treated with a 5.0‐fold molar excess of NaOH aq at room temperature (ca 25–27 °C) for 18 h.

**Scheme 1:**
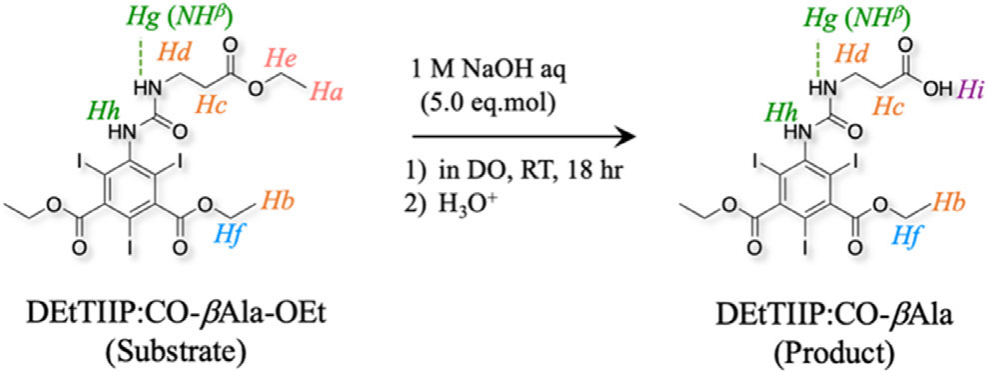
Selective hydrolysis of esters in DEtTIIP.

The recovered product was identified as DEtTIIP:CO‐*β*Ala (**Scheme** **1**) by ^1^H‐NMR (Figure 5B), since the signals assigned to the *β*Ala ethyl esters (*H*_*a*_, 1.20, t, 3H and He, 4.08, q, 2H) of the substrate disappeared, whereas those of the ethyl esters on the 1,3‐carbonyls of the isophthalate substructure were preserved as (*H*_*b*_, 1.35, t, 6H) and (*H*_*f*_, 4.36, q, 4H) (Figure 5B). The ^1^H chemical shifts (*δ*, ppm) of the C^*α*^ and C^*β*^ carbons in *β*Ala were (*H*_*c*_, 2.43, t, 2H) and (*H*_*d*_, 3.27, q, 2H), respectively, which were slightly lower than those found in the substrate (*H*_*c*_, 2.49, m, 2.9H; *H*_*d*_, 3.29, q, 2H). A new proton signal (*H*_*i*_, 12.25, s, 1H) was observed in the product spectrum, which was assigned to the carboxylic acid of *β*Ala.

In the ^13^C‐CPD/DEPT135‐NMR spectra (Figure 6, blue and green traces), the carbon signals of the ethyl ester on *β*Ala (*C*_*b*_, 14.6, upward; *C*_*e*_, 60.4, downward) were not found for the product, while other carbon signals were preserved, except for one signal of the *β*Ala carbonyl at 172.1 (*C*_*m*_) for the substrate, but at 173.7 (*C*_*n*_) for the product. The spectroscopic observations support the fact that the ethyl ester of *β*Ala was hydrolyzed, but the ethyl esters of 1,3‐isophthalate were resistant to hydrolysis even at a 5.0 eq.mol excess NaOH. The resistance of the 1,3‐ethyl isophthalate is due to the steric hindrance by the 2,4,6‐iodines, as shown in Figure 1B and 7A.

### 2.5 N‐Carbamoylation of Amino Acid Ester Using DAcOEtTIIP‐NCO

**Figure 8:**
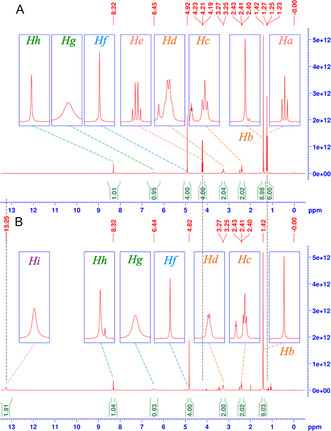
Comparison of ^1^H‐NMR spectra before and after the 2.4 eq.mol alkaline treatment of A) the DAcOEtTIIP:CO‐*β*Ala‐O^t^Bu substrate and B) the DAcOHTIIP:CO‐*β*Ala‐O^t^Bu product.

*tert*‐Butyl *β*Ala (*β*Ala‐O^t^Bu) monohydrochloride was selected as the first substrate to be tested for carbamoylation using DAcOEtTIIP‐NCO. The addition reaction at 60 °C for 16 h gave the product in 89 mol% yield. The product was identified as diacetoxyethyl 5‐(carbonyl‐*β*‐alanyloxy *tert*‐butyl)amino‐2,4,6‐triiodoisophthalate (DAcOEtTIIP:CO‐*β*Ala‐O^t^Bu) by ^1^H‐NMR (**Figure** **8**A): the ^1^H signals on the ethyl esters bound to the 1,3‐acetoxy groups of isophthalate (*H*_*a*_, methyl, 1.26, t, 6H; *H*_*e*_, methylene, 4.22, q, 4H), the C^*α*^ (*H*_*c*_, 2.41, t, 2H) and C^*β*^ (*H*_*d*_, 3.28, q, 2H) of *β*Ala, the methylene of 1,3‐acetoxy esters (*H*_*f*_, 4.92, s, 4H), and the urea substructure for *N* ^*β*^H (*H*_*g*_, 6.45, s, 1H) and *N* ^*5*^H (*H*_*h*_, 8.32, s, 1H).

**Figure 9:**
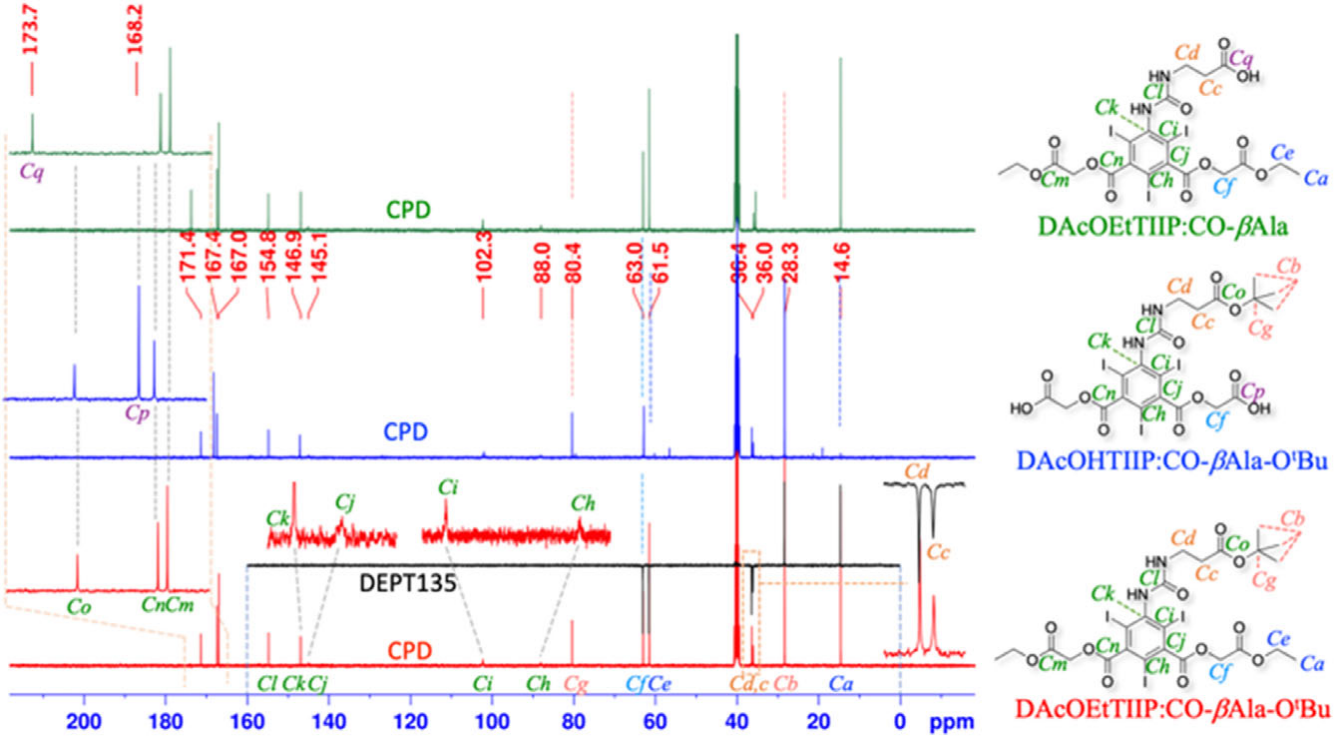
Comparison of ^13^C‐CPD/DEPT135‐NMR spectra before and after the 2.4 eq.mol alkaline treatment of the DAcOEtTIIP:CO‐*β*Ala‐O^t^Bu substrate (red and black traces) and the DAcOHTIIP:CO‐*β*Ala‐O^t^Bu product (blue trace). The treatment using excess TFA removed the ^t^Bu ester to give DAcOEtTIIP:CO‐*β*Ala (green trace), while preserving other substructures.

The carbon signals on the ^13^C‐CPD/DEPT135‐NMR (**Figure** **9**, red and black traces) of the product were also completely assigned as DAcOEtTIIP:CO‐*β*Ala‐O^t^Bu: the ethyl esters on the 1,3‐acetoxy groups of isophthalate (*C*_*a*_, 14.6, upward; *C*_*e*_, 61.5, downward), the C^*α*^ (*C*_*c*_, 36.0, downward) and C^*β*^ (*C*_*d*_, 36.4, downward) of *β*Ala, the methylene of the 1,3‐acetoxy esters (*C*_*f*_, 63.0, downward), the ^t^Bu ester (*C*_*b*_, 28.3; *C*_*g*_, 80.4), the aromatic carbons as C^2^ (*C*_*h*_, 88.0), C^4,6^ (*C*_*i*_, 102.3), C^1,3^ (*C*_*j*_, 145.1), and C^5^ (*C*_*k*_, 146.9), the urea carbonyl (*C*_*l*_, 154.8), the 1,3‐acetoxy ester carbonyl (*C*_*m*_, 167.0), the 1,3‐carbonyl of isophthalate (*C*_*n*_, 167.4), and the *β*Ala carbonyl (*C*_*o*_, 171.4).

Figure 7B represents the rank 1 conformation from the conformer search calculation, and the C5 and C6 views indicate that the nucleophilic center carbon (*) of the 5‐isocyanato group is not sterically hindered by the adjacent 2,6‐iodines and is active for the addition reaction between DAcOEtTIIP‐NCO and *β*Ala‐O^t^Bu.

### 2.6 Selective Conversion of Esters in DAcOEtTIIP:CO‐*β*Ala‐O^t^Bu to Carboxylic Acids

**Scheme 2:**
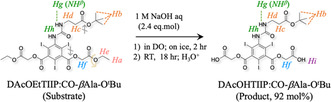
Alkaline hydrolysis of esters in DAcOEtTIIP:CO‐*β*Ala‐O^t^Bu.

Upon cooling, DAcOEtTIIP:CO‐*β*Ala‐O^t^Bu was treated with 2.4 eq.mol NaOH aq (1.2 eq.mol for each of the 1‐ or 3‐acetoyethyl esters on the DAcOEtTIIP substructure), and the product was recovered in 92 mol% yield (**Scheme** **2**).

The ^1^H‐NMR spectra (Figure 8B) indicated that the product was identified as 5‐(carbonyl‐*β*‐alanyloxy *tert*‐butyl)amino‐2,4,6‐triiodoisophthaloyl bis(oxy)diacetic acid (DAcOHTIIP:CO‐*β*Ala‐O^t^Bu), indicating that the 1,3‐diacetoxyethyl ester groups were selectively hydrolyzed to the corresponding 1,3‐diacetic acids, which is evident in the disappearance of the methyl (*H*_*a*_, 1.25, t, 6H) and methylene (*H*_*e*_, 4.22, q, 4H) signals found in the substrate. The 1,3‐diacetoxymethylene (*H*_*f*_, 4.92, s, 4H) of the substrate was preserved, whereas a new signal (*H*_*i*_, 13.3, s, 2H), assigned to the carboxylic proton, was found in the product spectrum. The carbon signals (Figure 9, blue trace) of the methyl (*C*_*a*_, 14.6) and methylene (*C*_*e*_, 61.5) of the substrate were not found in the ^13^C‐CPD/DEPT135‐NMR spectrum of the product, and the substrate chemical shift of the 1,3‐diacetoxy carbonyl (*C*_*m*_, Figure 9, red trace) was changed from 167.0 to 168.2 (*C*_*p*_, Figure 9, blue trace) after the alkaline hydrolysis.

This result, along with the resistance of the 1,3‐diethyl ester in DEtTIIP (Figures 5 and 6), clearly indicated that the 1,3‐carbonyl carbon of the 2,4,6‐triiodoisophthalate derivative was not active for the nucleophilic attack due to the steric hindrance by the adjacent iodines (Figure 1C), and in general, the ^t^Bu esters are not highly reactive to alkaline hydrolysis, so the selective hydrolysis of the 1,3‐diacetoxy ethyl ester proceeds to yield the product, DAcOHTIIP:CO‐*β*Ala‐O^t^Bu.

**Figure 10:**
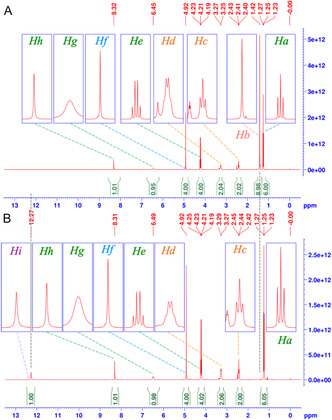
Comparison of ^1^H‐NMR spectra before and after the excess TFA treatment of A) the DAcOEtTIIP:CO‐*β*Ala‐O^t^Bu substrate and B) the DAcOEtTIIP:CO‐*β*Ala product.

**Scheme 3:**
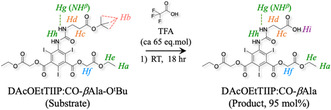
Selective fission of esters in DAcOEtTIIP:CO‐*β*Ala‐O^t^Bu.

On the other hand, when the substrate was treated with an excess amount of trifluoroacetic acid (TFA), the ^t^Bu ester of the substrate was rapidly removed to give the product, diacetoxyethyl 5‐(carbonyl‐*β*‐alanine)amino‐2,4,6‐triiodoisophthalate (DAcOEtTIIP:CO‐*β*Ala), as shown Figure 9B and **Figure** **10** (green trace), identified by the disappearances of the ^t^Bu proton signal (*H*_*b*_, 1.42, s, 9H) and the carbon signal (*C*_*g*_, 80.4) of the substrate and by development of the proton and carbon signals assigned as the carboxylic acid at 12.3 ppm (*H*_*i*_, s, 1H) and 173.7 ppm (*C*_*q*_), respectively (**Scheme** **3**).

### 2.7 Carbamoylation of *N* ^*ε*^‐Amino Group of Lys Derivative

**Figure 11:**
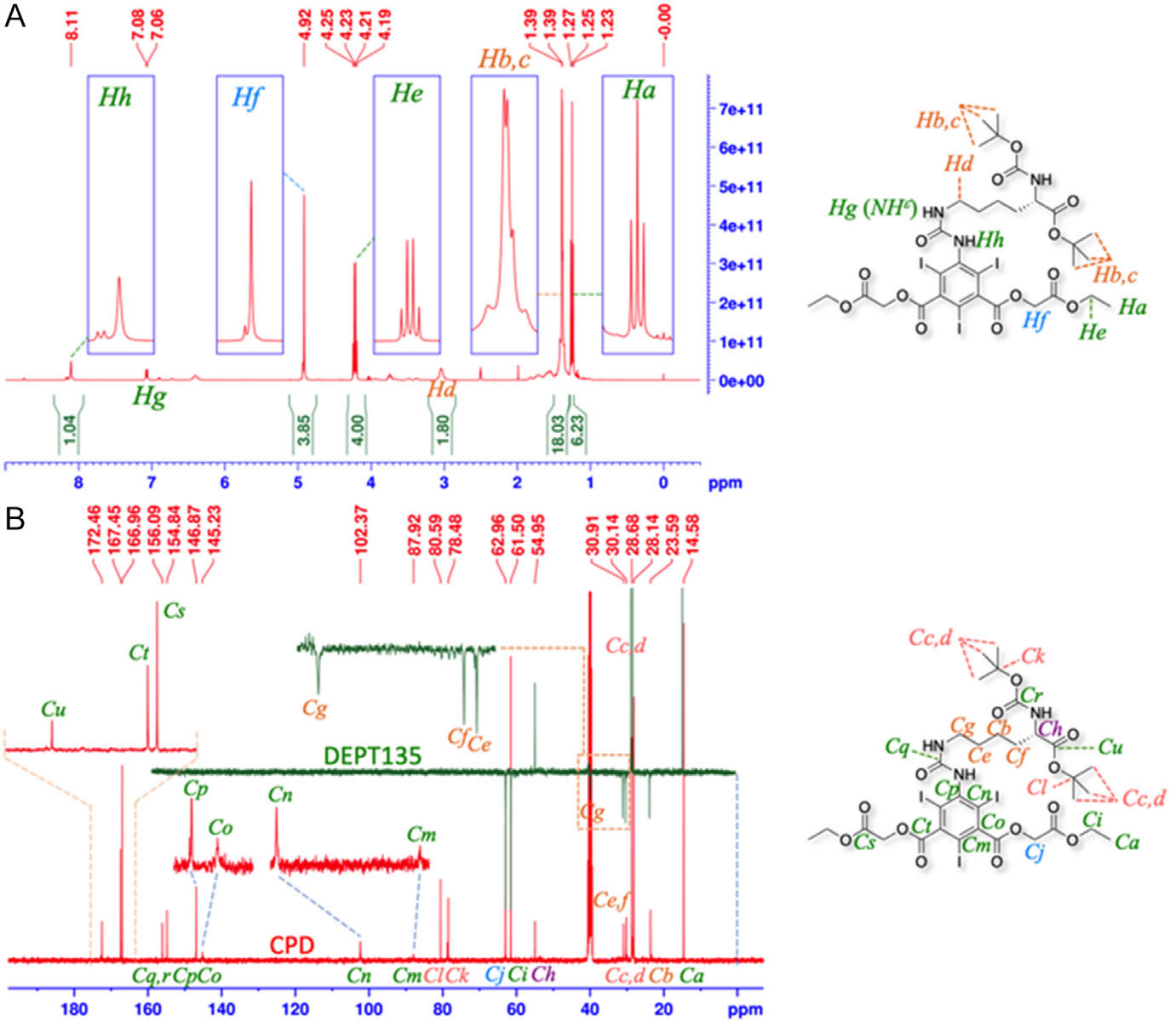
A) ^1^H‐ and B) ^13^C‐CPD//DEPT135 NMR spectra of Boc‐Lys(CO:DAcOEtTIIP)‐O^t^Bu.

As a model case for peptide or poly(amino acid) synthesis using DAcOEtTIIP‐NCO, *N* ^*α*^‐*tert*‐butyloxycarbonyl‐L‐lysine *tert*‐butyl ester (Boc‐Lys‐O^t^Bu) was selected as the second substrate. The addition reaction was performed at 60 °C for 18 h, and the product, Boc‐Lys(CO:DAcOEtTIIP)‐O^t^Bu, was recovered in 87 mol% yield. **Figure** **11**A represents the ^1^H‐NMR spectrum of the product, and the characteristic signals were found as follows: the methyl (*H*_*a*_, 1.25, t, 6H) and the methylene (*H*_*e*_, 4.22, q, 4H) of the 1,3‐diacetoxy ethyl group; two ^t^Bu groups (*H*_*b*_ *,c*, ss, 18H) in *N* ^*α*^‐Boc and the ‐O^t^Bu of the Lys substructure; the acetoxymethylene (*H*_*f*_, 4.92, s, 4H); and the urea substructure (*H*_*g*_, *N* ^*ε*^H, 7.07, ss, 1H; *H*_*h*_, *N* ^*5*^H, 8.11, s, 1H).

All of the carbon signals found in the ^13^C‐CPD/DEPT135‐NMR spectrum (Figure 11B) were assigned as follows: the methyl (*C*_*a*_, 14.6, upward) and the methylene (*C*_*i*_, 61.5, downward) of the 1,3‐diacetoxy ethyl group; the Lys C^*α*^ (*C*_*h*_, 55.0) and the side chain carbons, C^*β*^ (*C*_*f*_, 30.1, downward), C^*γ*^ (*C*_*b*_, 23.6, downward), C^*δ*^ (*C*_*e*_, 30.9, downward), C^*ε*^ (*C*_*g*_, 40.1, downward); the primary (*C*_*c, d*_; 28.1, 28.7, upward) and tertiary (*C*_*k*_, 78.5; Cl, 80.6) carbons of two ^t^Bu groups; the aromatic carbons, C^2^ (*C*_*m*_, 87.9), C^4,6^ (*C*_*n*_, 102.4), C^1,3^ (*C*_*o*_, 145.2), C^5^ (*C*_*p*_, 146.9); the urea carbonyl (*C*_*q*_, 154.8); the Boc carbonyl (*C*_*r*_, 156.1); the 1,3‐diacetoxy carbonyls (*C*_*s*_, 166.0); the 1,3‐carbonyls (*C*_*t*_, 167.5); the Lys *α*‐carbonyl (*C*_*u*_, 172.5).

As described in the previous section, the Boc‐ and ‐O^t^Bu groups are removable *via* a one‐pot reaction using TFA, and the product side chain is still protected by the 1,3‐diacetoxy esters, which means that the product is a precursor of the *N*‐carboxy anhydride monomer of the modified Lys residue to be used in the poly(amino acid)s synthesis.

## 3 Conclusion

Chemical modification of ATIIPA involves a broad spectrum of the radiopaque or imaging contrast agent developments, such as multimerization of the ATTIPA substructures,^[^ ^9^ ^,^ ^10^ ^]^ the cationic derivative for the quantitative cartilage visualization,^[^ ^18^ ^,^ ^19^ ^]^ the polymer foams of ATIIPA for aneurysm occlusion,^[^ ^20^ ^]^ the biodegradable CT‐contrast comprising the ATIIPA derivative,^[^ ^21^ ^]^ or the fullerene‐iohexol conjugate for X‐ray imaging.^[^ ^22^ ^,^ ^23^ ^]^ Strategies for the chemical modification of ATIIPA have mostly adopted the conversion of the 1,3‐dicarboxyl groups to the corresponding amides *via* the 1,3‐carboxylic chlorides, in which the ATIIPA derivatives are used as acylating agents. In contrast, in this study, ATIIPA was used as a nucleophile for conversion to the corresponding esters, and the resulting ATIIPA esters were tested as *N*‐carbamoylation agents for the amino acid esters.

ATIIPA is the precursor compound of DEtTIIP and DAcOEtTIIP and contains 5‐amino and 1,3‐carboxyl groups. Therefore, ATIIPA is one of the amino acids in a broad sense. In the typical strategy of peptide synthesis, the *N* ^*α*^‐protected amino acid (the acid component) and *N* ^*α*^‐free amino acid ester (the amino component) are condensed using the coupling reagents in which the acid component acts as a *N* ^*α*^‐acylating agent toward the amino component. When ATIIPA is recognized as an amino acid‐type building block, the 5‐amino and the 1,3‐carboxylic acids should be protected during certain steps of the synthesis. However, the 1,3‐carboxyl groups of ATIIPA are highly hindered by the adjacent iodines, whereas they are sufficiently reactive for protection (*O*‐alkylation using the halides) only by the method using the base‐catalyzed esterification.

For the resulting ATIIPA diesters, as seen in the case of DEtTIIP, the removal of the diethyl esters by alkaline hydrolysis was difficult because of the steric hindrance of the 1,3‐carbonyls. DAcOEtTIIP:CO‐*β*Ala‐O^t^Bu can thus provide a route for deprotection by the alkaline hydrolysis to give DAcOHTIIP:CO‐*β*Ala‐O^t^Bu, because of its accessible 1,3‐diacetoxythyl esters. On the other hand, the TFA treatment gives the 1,3‐acetoxyethyl protected derivative DAcOEtTIIP:CO‐*β*Ala, which can be further used as an *N*‐acylating agent. A model compound, Boc‐Lys(CO:DAcOEtTIIP)‐O^t^Bu, is also chemically orthogonal to the TFA treatment for removal of the Boc and ^t^Bu ester protections along with preserving the 1,3‐diacetoxyethyl protection, or to the alkaline hydrolysis for removal of the 1,3‐diacetoxyethyl protection while maintaining the Boc‐ and ^t^Bu ester protections.

In conclusion, DEtTIIP and DAcOEtTIIP are multipurpose carbamoylation components via isocyanation, and the adducts of the corresponding isocyanate and the amino acid esters can lead to the chemical structure being adopted for the selective deprotection, which is feasible in general organic synthesis, for example, the bioconjugates or the conjugated peptides having the radiopaque properties due to the 2,4,6‐triiodine substituents. This study thus provides a feasible concept for use of the ATIIPA esters as a building block of organic synthesis under mild reaction conditions, which potentially connects to the development of a novel medical device^[^ ^24^ ^]^

## 4 Experimental Section

4.1

4.1.1

##### Materials

All chemicals used in this study were of reagent grade or special grade, as defined by the manufacturers (90–95%GC). 5‐Amino‐2,4,6‐triiodoisophthalic acid (ATIIPA), *β*‐alanine *tert*‐butyl ester monohydrochloride (HCl *β*Ala‐O^t^Bu), and ethyl *α*‐bromoacetate were purchased from the Tokyo Chemical Industry (TCI, Tokyo, Japan). Bromoethane, triethylamine (TEA), and absolute solvents of organic synthesis grade were obtained from the Wako‐Fujifilm Pure Chemical Industry (Osaka, Japan).

The *β*‐alanine ethyl ester monohydrochloride (HCl *β*Ala‐OEt) was prepared by a typical esterification of the amino acids. Briefly, into a suspension of *β*‐alanine in ethanol, thionyl chloride (3.6 eq.mol) was added dropwise upon cooling using an ice bath, and the reaction mixture was heated to 70–80 °C until complete dissolution of the *β*Ala, followed by a series of workups including evaporation of the solvent, crystallization of the crude product by adding diethyl ether, and recrystallization from ethanol and diethyl ether.

##### Conformer Search Calculation

CONFLEX software version 7.x (CONFLEX, Tokyo, Japan)^[^ ^25^ ^,^ ^26^ ^]^ was employed to calculate the conformer population percentages (CP%),^[^ ^27^ ^,^ ^28^ ^]^ based on the steric energies.^[^ ^29^ ^]^ The calculations were typically performed under MMFF94s force fields^[^ ^30^ ^]^ and the full‐matrix Newton‐Rapson (FMNR) convergence method to find the steric energy with the search limit of 4.0 kcal mol^–1^ (no solvent effects). The top‐populated conformers were visualized using the CONFLEX built‐in function or the Avogadro software.^[^ ^31^ ^]^

##### Infrared Spectroscopy

A Horiba FT720 spectrophotometer (Kyoto, Japan) was used to record the infrared spectra of the samples, DEtTIIP, DAcOEtTIIP, and their corresponding isocyanates. The crystalline DEtTIIP and DAcOEtTIIP samples were prepared as KBR pellets (phi, 8 mm), and the oily isocyanates were spread on a single‐crystal NaCl prism (thickness, 3.5 mm) for the transmission spectra acquisition.

##### Nuclear Magnetic Resonances

The samples (ca. 30–60 mg each) were dissolved in dimethyl sulfoxide (DMSO)‐*d*6 containing 0.05% tetramethylsilane (TMS) and transferred to a conventional NMR tube. A Bruker AVANCE NEO (Bruker Japan, Tokyo) was employed for spectral recordings with the built‐in pulse programs for ^1^H‐NMR, the complete pulse decoupling (CPD) ^13^C‐NMR, and the distortionless enhancement by polarization transfer (DEPT) 135 ^13^C‐NMR. Data visualization and analysis were carried out using TopSpin Software provided by Bruker (Figure 6, 8, 9, 10, 11) or by the author's original Python script (Figures S1B and 5, Supporting Information), importing the nmrglue library.^[^ ^32^ ^]^

##### Synthesis

**DEtTIIP:** ATIIPA (5.00 g; 8.95 mmol; 1.00 eq.mol) was dissolved in absolute DMF (20 mL), and NaHCO_3_ (3.16 g; 37.6 mmol; 4.2 eq.mol) was added to the solution. After the evolution of CO_2_ gas had stopped, bromoethane (4.18 mL; 5.85 g; 53.7 mmol; 6.00 eq.mol) was mixed with the suspension, and the reaction was continued for 18 h at 45 °C. The reaction mixture was then diluted with ethyl acetate (EtOAc, 180 mL), and the organic layer was washed three times with aqueous NaHCO_3_ (5.0 (w/v)%) and brine and subsequently dried over anhydrous Na_2_SO_4_. After evaporation of the solvent under reduced pressure, the residue was crystallized with *n*‐hexane and recovered by filtration. The crude product was recrystallized from ethyl acetate (EtOAc) and *n*‐hexane. Yield, 5.20 g (95.4%). DEtTIIP: (^1^H‐NMR, 400 MHz, DMSO*d*6): *δ* (ppm, TMS): 1.34 (t, 6H, *J* = 7.12 Hz, *H*_*a*_, 1,3‐COCH_2_CH_3_), 4.34 (q, 4H, *J* = 7.11 Hz, *H*_*b*_, 1,3‐COCH_2_CH_3_), 5.68 (s, 2H, *H*_*c*_, 5‐NH_2_); (^13^C‐NMR, 101 MHz, DMSO*d*6): *δ* (ppm, TMS): 14.28 (*C*_*a*_, 1,3‐CO‐CH_2_ CH_3_), 62.58 (*C*_*b*_, 1,3‐CO‐CH_2_CH_3_), 71.74 (*C*_*c*_, C ^2^‐I), 79.93 (*C*_*d*_, C ^4,6^‐I), 146.93 (*C*_*e*_, C ^1,3^‐CO‐), 148.67 (*C*_*f*_, C ^5^‐NH_2_), 168.51 (*C*_*g*_, 1,3‐COOEt). See Figure S1 and S2, Supporting Information, for FTIR and ^1^H‐/^13^C‐CPD NMR spectra, respectively.

**DAcOEtTIIP:** ATIIPA (2.00 g; 3.58 mmol; 1.00 eq.mol) was dissolved in absolute DMF (15 mL), and NaHCO_3_ (2.65 g; 13.6 mmol; 3.8 eq.mol) was added to the solution. After the evolution of CO_2_ gas stopped, *tert*‐butyl *α*‐bromoacetate (1.99 mL; 2.65 g; 13.6 mmol; 3.80 eq.mol) was mixed with the suspension, and the reaction was continued for 18 h at 45 °C. The reaction mixture was then diluted with ethyl acetate (EtOAc, 60 mL), and the organic layer was washed three times with aqueous NaHCO_3_ (5.0 (w/v)%) and brine and subsequently dried over anhydrous Na_2_SO_4_. After evaporation of the solvent under reduced pressure, the residue was crystallized with *n*‐hexane and recovered by filtration. The crude product was recrystallized from ethyl acetate (EtOAc) and *n*‐hexane. Yield, 2.60 g (92.3%). DAcOEtTIIP: (^1^H‐NMR, 400 MHz, DMSO*d*6): *δ* (ppm, TMS): 1.25 (t, 6H, *J* = 7.11 Hz, *H*_*a*_, 1,3‐COCH_2_CO‐CH_2_CH_3_), 4.22 (q, 4H, *J* = 7.11 Hz, *H*_*b*_, 1,3‐COCH_2_CO‐CH_2_CH_3_), 4.90 (s, 4H, *H*_*c*_, 1,3‐COCH_2_CO‐CH_2_CH_3_), 5.77 (s, 2H, *H*_*d*_, 5‐NH_2_); (^13^C‐NMR, 101 MHz, DMSO*d*6): *δ* (ppm, TMS): 14.59 (*C*_*a*_, 1,3‐COCH_2_COOCH_2_ CH_3_), 61.49 (*C*_*b*_, 1,3‐COCH_2_COCH_2_‐CH_3_), 62.85 (*C*_*c*_, 1,3‐COCH_2_COOCH_2_CH_3_), 71.95 (*C*_*d*_, C ^2^‐I), 80.70 (*C*_*e*_, C ^4,6^‐I), 146.14 (*C*_*f*_, C ^1,3^‐CO‐), 148.94 (*C*_*g*_, C ^5^‐NH‐), 167.05 (*C*_*h*_, 1,3‐COCH_2_COOEt), 167.86 (*C*_*i*_, 1,3‐COCH_2_ COOEt).

**DEtTIIP‐NCO:** DEtTIIP (1.50 g; 2.44 mmol; 1.00 eq.mol) was dissolved in absolute 1,4‐dioxane (10 mL). To this solution added were dried charcoal powder (0.05 g) and bis(trichloromethyl) carbonate (TCMC, 2.17 g; 3.00 eq.mol of COCl_2_ in situ); then, the reaction was continued for 6 h at 75 °C. After annealing the reaction mixture, the charcoal powder was removed by filtration, and the solvent and excess COCl_2_ were evaporated under reduced pressure. The residue was completely dried in vacuo for 2 h at 55 °C. After confirmation of the NCO stretching vibration at 2264 cm^−1^ by infrared spectroscopy, the oily product was immediately used for the carbamoylation reactions. Yield, 1.55 g (99.1 mol%). See Figure S3, Supporting Information, for FTIR soectral change.

**DAcOEtTIIP‐NCO:** DAcOEtTIIP (2.00 g; 2.74 mmol; 1.00 eq.mol) was dissolved in absolute 1,4‐dioxane (15 mL). To this solution added were dried charcoal powder (0.05 g) and TCMC (0.81 g; 2.74 mmol; 3.00 eq.mol of COCl_2_ in situ), then the reaction was continued for 6 h at 75 °C. After annealing the reaction mixture, filtration for removal of the charcoal powder, evaporation of solvent, and excess COCl_2_ under reduced pressure, the residue was completely dried at 65 °C in vacuo for 2 h and subjected to infrared spectrum recording using a single‐crystal NaCl plate to confirm the NCO stretching vibration at 2264 cm^−1^. The product was immediately used for the carbamoylation reactions. Yield, 2.05 g (99.0 mol%).

**DEtTIIP:CO‐*β*Ala‐OEt:** DETIIP‐NCO (1.50 g; 2.44 mmol; 1.00 eq.mol) was dissolved in absolute 1,4‐dioxane (6.00 mL), and to this solution added were HCl·*β*Ala‐OEt (0.40 g; 2.54 mmol; 1.10 eq.mol) and triethylamine (TEA, 360 µL; 0.26 g; 2.57 mmol; 1.10 eq.mol), and the reaction was continued for 18 h at 70 °C. The reaction mixture was diluted with EtOAc (60 mL) and the organic layer was washed twice with 10 (w/v)% aqueous citrate (30 mL) and brine (30 mL), followed by dehydration over Na_2_SO_4_. After evaporation of the solvent, the residue spontaneously crystallized and was dispersed in *n*‐hexane for recovery by filtration. The crude product was recrystallized from ethyl acetate (EtOAc) and *n*‐hexane. Yield, 1.54 g (87.4 mol%). DEtTIIP:CO‐*β*Ala‐OEt: (^1^H‐NMR, 400 MHz, DMSO*d*6): *δ* (ppm, TMS): 1.20 (t, 3H, *J* = 7.11 Hz, *H*_*a*_, ‐*β*Ala‐OCH_2_CH_3_), 1.34 (t, 6H, *J* = 7.11 Hz, *H*_*b*_, 1,3‐COOCH_2_CH_3_), 2.49 (t, 2H, *J* = 6.06 Hz, *H*_*c*_, ‐C^*α*^ H_2_‐ of *β*Ala), 3.29 (q, 2H, *J* = 6.18 Hz, *H*_*d*_, ‐C^*β*^ H_2_‐ of *β*Ala), 4.08 (q, 2H, *J* = 7.11 Hz, *H*_*e*_, ‐*β*Ala‐OCH_2_CH_3_), 4.36 (q, 4H, *J* = 7.08 Hz, *H*_*f*_, 1,3‐COOCH_2_CH_3_), 6.48 (s, 1H, *H*_*g*_, 5‐NHCONH‐*β*Ala‐), 8.25 (s, 1H, *H*_*h*_, 5‐NHCONH‐*β*Ala‐); (^13^C‐NMR, 101 MHz, DMSO*d*6): *δ* (ppm, TMS): 14.23 (*C*_*a*_, 1,3‐COOCH_2_ CH_3_), 14.61 (*C*_*b*_, ‐*β*Ala‐OCH_2_ CH_3_), 35.27 (*C*_*c*_, C ^*α*^ of *β*Ala), 35.92 (*C*_*d*_, C ^*β*^ of *β*Ala), 60.45 (*C*_*e*_, ‐*β*Ala‐OCH_2_CH_3_), 62.81 (*C*_*f*_, 1,3‐COOCH_2_CH_3_), 87.63 (*C*_*g*_, C ^2^‐I), 101.23 (*C*_*h*_, C ^4,6^‐I), 144.66 (*C*_*i*_, C ^1,3^‐CO‐), 147.65 (*C*_*j*_, C ^5^‐NH‐), 154.88 (*C*_*k*_, 1,3‐COOCH_2_CH_3_), 168.10 (*C*_*l*_, 5‐NHCONH‐*β*Ala), 172.07 (*C*_*m*_, ‐*β*Ala‐COOEt).

**DEtTIIP:CO‐*β*Ala:** DEtTIIP:CO‐*β*Ala‐OEt (0.40 g; 528 µmol; 1.00 eq.mol) was dissolved in 1,4‐dioxane (5.00 mL) and a 1.00 M NaOH aqueous solution (2.64 mL; 2.64 mmol; 5.00 eq.mol) was dropwise added at room temperature, and the reaction was continued for 18 h. The reaction mixture was then condensed under reduced pressure, and the residue was dispersed in distilled water (40 mL) and transferred into a separation funnel for acidification using a 1.0 M HCl aqueous solution (3.27 mL; 3.17 mmol; 6.00 eq.mol), confirming pH 2.0 or below. The precipitate was extracted twice with EtOAc (20 mL), and the combined organic phase was washed twice with brine (30 mL) for neutralization, followed by dehydration over Na_2_SO_4_. After evaporation of the solvent, the residue was crystallized by pouring *n*‐hexane and subsequently recovered by filtration. The crude product was dissolved in EtOAc (10 mL) and the product solute was reversibly extracted with 5.0 (w/v)% NaHCO_3_, and the combined aqueous phase was acidified to pH 2.5, followed by extraction of the precipitate with EtOAc (15 mL). The organic phase was washed with brine for neutralization and dried over Na_2_SO_4_. The purified product was recovered by evaporation in vacuo and recrystallized from EtOAc and *n*‐hexane. Yield, 0.37 g (96.1 mol%). DEtTIIP:CO‐*β*Ala: (^1^H‐NMR, 400 MHz, DMSO*d*6): *δ* (ppm, TMS): 1.35 (t, 6H, *J* = 7.12 Hz, *H*_*b*_, 1,3‐COOCH_2_CH_3_), 2.43 (t, 2H, *J* = 6.61 Hz, *H*_*c*_, ‐C^*α*^ H_2_‐ of *β*Ala), 3.27 (q, 2H, *J* = 6.12 Hz, *H*_*d*_, ‐C^*β*^ H_2_‐ of *β*Ala), 4.36 (q, 4H, *J* = 7.07 Hz, *H*_*f*_, 1,3‐COOCH_2_CH_3_), 6.47 (s, 1H, *H*_*g*_, 5‐NHCONH‐*β*Ala), 8.27 (s, 1H, *H*_*h*_, 5‐NHCONH‐*β*Ala), 12.25 (s, 1H, *H*_*i*_, ‐*β*Ala‐COOH); (^13^C‐NMR, 101 MHz, DMSO*d*6): *δ* (ppm, TMS): 14.25 (*C*_*b*_, 1,3‐COOCH_2_ CH_3_), 35.38 (*C*_*c*_, C ^*α*^ of *β*Ala), 35.91 (*C*_*d*_, C ^*β*^ of *β*Ala), 62.79 (*C*_*f*_, 1,3‐COOCH_2_CH_3_), 87.60 (*C*_*g*_, C ^2^‐I), 101.26 (*C*_*h*_, C ^4,6^‐I), 144.73 (*C*_*i*_, C ^1,3^‐CO‐), 147.63 (*C*_*j*_, C ^5^‐NH‐), 154.88 (*C*_*k*_, 1,3‐COOCH_2_CH_3_), 168.09 (*C*_*l*_, 5‐NHCONH‐*β*Ala), 173.74 (*C*_*n*_, ‐*β*Ala‐COOH).

**DAcOEtTIIP:CO‐*β*Ala‐O** ^**t**^ **Bu:** DAcOEtTIIP‐NCO (2.07 g, 2.74 mmol, 1.00 eq mol) was dissolved in absolute 1,4‐dioxane (8.00 mL). To this solution added were *β*Ala‐O^t^Bu monohydrochloride (0.60 g, 3.28 mmol, 1.20 eq.mol) and TEA (458 µL, 0.33 g, 3.28 mmol, 1.20 eq.mol), and the reaction was continued for 16 h at 60 °C. The reaction mixture was diluted with EtOAc (70 mL) and the organic layer was washed twice with aqueous HCl (200 mM, 40 mL) and brine (50 mL), followed by dehydration over Na_2_SO_4_. After evaporation of the solvent, the residue was crystallized in *n*‐hexane for recovery by filtration and subsequent drying. The crude product was recrystallized from ethyl acetate (EtOAc) and *n*‐hexane. Yield, 2.21 g (89.3 mol%). DAcOEtTIIP‐CO:*β*Ala‐OtBu: (^1^H‐NMR, 400 MHz, DMSO*d*6): *δ* (ppm, TMS): 1.25 (t, 6H, *J* = 7.11 Hz, *H*_*a*_, 1,3‐COCH_2_COOCH_2_CH_3_), 1.42 (s, 9H, *H*_*b*_, ‐*β*Ala‐OC(CH_3_)_3_), 2.41 (t, 2H, *J* = 6.80 Hz, *H*_*c*_, C^*α*^ H_2_ of *β*Ala), 3.26 (q, 2H, *J* = 6.12 Hz, *H*_*d*_, C^*β*^ H_2_ of *β*Ala), 4.22 (q, 4H, *J* = 7.11 Hz, *H*_*e*_, 1,3‐COCH_2_COOCH_2_CH_3_), 4.92 (s, 4H, *H*_*f*_, 1,3‐COCH_2_COOCEt), 6.45 (s, 1H, *H*_*g*_, 5‐NHCONH‐*β*Ala‐), 8.32 (s, 1H, *H*_*h*_, 5‐NHCONH‐); (^13^C‐NMR, 101 MHz, DMSO*d*6): *δ* (ppm, TMS): 14.58 (*C*_*a*_, 1,3‐COCH_2_COOCH_2_ CH_3_), 28.31 (*C*_*b*_, ‐*β*Ala‐OC(CH_3_)_3_), 36.04 (*C*_*c*_, C ^*α*^ of *β*Ala), 36.38 (*C*_*d*_, C ^*β*^ of *β*Ala), 61.51 (*C*_*e*_, 1,3‐COCH_2_COOCH_2_CH_3_), 62.98 (*C*_*f*_, 1,3‐COCH_2_COOCH_2_CH_3_), 80.42 (*C*_*g*_, ‐*β*Ala‐OC(CH_3_)_3_), 88.02 (*C*_*h*_, C ^2^‐I), 102.25 (*C*_*i*_, C ^4,6^‐I), 145.07 (*C*_*j*_, C ^1,3^‐CO‐), 146.92 (*C*_*k*_, C ^5^‐NH‐), 154.81 (*C*_*l*_, 5‐NHCONH‐*β*Ala‐), 166.98 (*C*_*m*_, 1,3‐COCH_2_ COOEt), 167.44 (*C*_*n*_, 1,3‐COCH_2_COOEt), 171.40 (*C*_*o*_, 5‐NHCONH‐CH_2_CH_2_ CO–O^t^Bu).

**DAcOHTIIP:CO‐*β*Ala‐O** ^**t**^ **Bu:** DAcOEtTIIP:CO‐*β*Ala‐O^t^Bu (0.30 g; 333 µmol 1.00 eq.mol) was dissolved in 1,4‐dioxane (3.5 mL). Upon cooling in an ice bath, an aqueous solution of NaOH (1.0 mol L^–^^1^; 0.40 mL; 2.4 eq.mol) was added dropwise (5 min). The reaction was continued for 1 h with cooling and additional 1 h at room temperature (ca. 26 °C). The reaction mixture was diluted with distilled water (20 mL), and in a separating funnel, 1 M HCl (900 µL) was dispensed to confirm the pH at 3.5, followed by two times of extractions with diethyl ether (Et_2_O, 10 mL). The combined organic layer was washed three times with brine (30 mL), followed by dehydration over Na_2_SO_4_. After evaporation of the solvent, the residue was dissolved in chloroform (6.00 mL) and EtOAc (2.5 mL) with heating at 60 °C. Upon annealing, seed crystals were formed and then *n*‐hexane (8.00 mL) was gradually added for complete crystallization. Yield, 0.26 g (92.4 mol%). DAcOHTIIP:CO‐*β*Ala‐O^t^Bu: (^1^H‐NMR, 400 MHz, DMSO*d*6): *δ* (ppm, TMS): 1.42 (s, 9H, *H*_*b*_, ‐*β*Ala‐OC(CH_3_)_3_), 2.41 (t, 2H, *J* = 6.76 Hz, *H*_*c*_, C^*α*^ H_2_ of *β*Ala), 3.26 (d, 2H, *J* = 5.99 Hz, *H*_*d*_, C^*β*^ H_2_ of *β*Ala), 4.82 (s, 4H, *H*_*f*_, 1,3‐COCH_2_COOH), 6.44 (s, 1H, *H*_*g*_, 5‐NHCONH‐*β*Ala‐), 8.32 (s, 1H, *H*_*h*_, 5‐NHCONH‐), 13.25 (s, 2H, *H*_*i*_, 1,3‐COCH_2_COOH); (^13^C‐NMR, 101 MHz, DMSO*d*6): *δ* (ppm, TMS): 28.32 (*C*_*b*_, ‐*β*Ala‐OC(CH_3_)_3_), 36.03 (*C*_*c*_, C ^*α*^ of *β*Ala), 36.37 (*C*_*d*_, C ^*β*^ of *β*Ala), 62.87 (*C*_*f*_, 1,3‐COCH_2_COOCH_2_CH_3_), 80.43 (*C*_*g*_, ‐*β*Ala‐OC(CH_3_)_3_), 88.04 (*C*_*h*_, C ^2^‐I), 102.04 (*C*_*i*_, C ^4,6^‐I), 144.98 (*C*_*j*_, C ^1,3^‐CO‐), 147.10 (*C*_*k*_, C ^5^‐NH‐), 154.83 (*C*_*l*_, 5‐NHCONH‐*β*Ala‐), 167.48 (*C*_*n*_, 1,3‐COCH_2_COOH), 168.24 (*C*_*p*_, 1,3‐COCH_2_ COOH), 171.40 (*C*_*o*_, 5‐NHCONH‐CH_2_CH_2_ CO–O^t^Bu).

**DAcOEtTIIP:CO‐*β*Ala:** DAcOEtTIIP:CO‐*β*Ala‐O^t^Bu (0.16 g; 177 µmol; 1.00 eq.mol) was dissolved in trifluoroacetic acid (TFA, 2.05 mL; 3.04 g; 26.6 mmol; 150 eq.mol). The reaction was continued for 3 h at room temperature (ca 26 °C). After evaporation of TFA, the crystalline residue was dispersed in anhydrous *n*‐hexane and recovered by filtration, followed by drying in vacuo at 50 °C. Yield, 0.14 g (93.3 mol%). DAcOEtTIIP:CO‐*β*Ala: (^1^H‐NMR, 400 MHz, DMSO*d*6): *δ* (ppm, TMS): 1.25 (t, 6H, *J* = 7.11 Hz, *H*_*a*_, 1,3‐COCH_2_COOCH_2_CH_3_), 2.44 (t, 2H, *J* = 6.62 Hz, *H*_*c*_, C^*α*^ H_2_ of *β*Ala), 3.28 (d, 2H, *J* = 6.01 Hz, *H*_*d*_, C^*β*^ H_2_ of *β*Ala), 4.22 (q, 4H, *J* = 7.11 Hz, *H*_*e*_, 1,3‐COCH_2_COOCH_2_CH_3_), 4.92 (s, 4H, *H*_*f*_, 1,3‐COCH_2_COOCEt), 6.49 (s, 1H, *H*_*g*_, 5‐NHCONH‐*β*Ala‐), 8.31 (s, 1H, *H*_*h*_, 5‐NHCONH‐), 12.27 (s, 1H, *H*_*i*_, ‐*β*Ala‐COOH); (^13^C‐NMR, 101 MHz, DMSO*d*6): *δ* (ppm, TMS): 14.59 (*C*_*a*_, 1,3‐COCH_2_COOCH_2_ CH_3_), 35.38 (*C*_*c*_, C ^*α*^ of *β*Ala), 35.93 (*C*_*d*_, C ^*β*^ of *β*Ala), 61.52 (*C*_*e*_, 1,3‐COCH_2_COOCH_2_CH_3_), 62.98 (*C*_*f*_, 1,3‐COCH_2_COOCH_2_CH_3_), 88.00 (*C*_*h*_, C ^2^‐I), 102.27 (*C*_*i*_, C ^4,6^‐I), 145.10 (*C*_*j*_, C ^1,3^‐CO‐), 146.89 (*C*_*k*_, C ^5^‐NH‐), 154.84 (*C*_*l*_, 5‐NHCONH‐*β*Ala‐), 166.99 (*C*_*m*_, 1,3‐COCH_2_ COOEt), 167.45 (*C*_*n*_, 1,3‐COCH_2_COOEt), 173.75 (*C*_*q*_, ‐*β*Ala‐COOH).

**Boc‐Lys(CO:DAcOEtTIIP)‐O** ^**t**^ **Bu:** DAcOEtTIIP‐NCO (1.04 g; 1.37 mmol; 1.00 eq.mol) was dissolved in absolute 1,4‐dioxane (5.00 mL) and 1.0 M Boc‐Lys‐O^t^Bu in an absolute 1,4 dioxane solution (1.44 mL; 1.44 mmol; 1.05 eq.mol) was added, and the reaction was continued at 60 °C for 18 h. The reaction mixture was diluted with EtOAc (50 mL) and the organic layer was washed two times with 10 (w/v)% citrate (30 mL) and (30 mL), followed by dehydration over Na_2_SO_4_. After the solvent was evaporated, the residue was washed with *n*‐hexane and dried in vacuo at 50 °C. Purification was performed by silica gel chromatography using EtOAc:*n*‐hexane = 70:30 as the mobile phase. Yield, 1.26 g (86.9 mol%). Boc‐Lys(DAcOEtTIIP:CO)‐O^t^Bu: (^1^H‐NMR, 400 MHz, DMSO*d*6): *δ* (ppm, TMS): 1.25 (t, 6H, *J* = 7.11 Hz, *H*_*a*_, 1,3‐COCH_2_COOCH_2_CH_3_), 1.39 (d, 18H, *J* = 1.92 Hz, *H*_*b, c*_, ‐OC(CH_3_)_3_ of Boc‐/‐O^t^Bu), 1.65 (m, 4H, *J* = 10.89 Hz, C^*β*^ H_2_ /C^*γ*^ H_2_ of ‐Lys‐), 3.04 (d, 2H, *J* = 4.79 Hz, *H*_*d*_, ‐C^*ε*^ H_2_‐), 4.22 (q, 4H, *J* = 7.11 Hz, *H*_*e*_, 1,3‐COCH_2_COOCH_2_CH_3_), 4.92 (s, 4H, *H*_*f*_, 1,3‐COCH_2_COOCEt), 7.07 (d, 1H, *J* = 7.71 Hz, *H*_*g*_, 5‐NHCONH‐C^*ε*^ of ‐Lys‐), 8.11 (s, 1H, *H*_*h*_, 5‐NHCONH‐); (^13^C‐NMR, 101 MHz, DMSO*d*6): *δ* (ppm, TMS): 14.58 (*C*_*a*_, 1,3‐COCH_2_COOCH_2_ CH_3_), 23.59 (*C*_*b*_, C ^*γ*^ of ‐Lys‐), 28.14 (*C*_*c*_, ‐OC(CH_3_)_3_ of Boc‐ or ‐O^t^Bu), 28.68 (*C*_*d*_, ‐OC(CH_3_)_3_ of Boc‐ or ‐O^t^Bu), 30.14 (*C*_*e*_, C ^*δ*^ of ‐Lys‐), 30.91 (*C*_*f*_, C ^*β*^ of ‐Lys‐), 54.95 (*C*_*g*_, C ^*ε*^ of ‐Lys‐), 61.50 (*C*_*h*_, C ^*α*^ of ‐Lys‐), 62.96 (*C*_*i*_, 1,3‐COCH_2_COOCH_2_CH_3_), 78.48 (*C*_*j*_, 1,3‐COCH_2_COOCH_2_CH_3_), 80.59 (*C*_*k*_, ‐OC(CH_3_)_3_ of Boc‐), 87.92 (*C*_*l*_, ‐OC(CH_3_)_3_ of ‐O^t^Bu), 102.37 (*C*_*m*_, C ^2^‐I), 145.23 (*C*_*n*_, C ^4,6^‐I), 146.87 (*C*_*o*_, C ^1,3^‐CO‐), 153.55 (*C*_*p*_, C ^5^‐NH‐), 154.84 (*C*_*q*_, 5‐NHCONH‐C^*ε*^ of ‐Lys), 156.09 (*C*_*r*_, ‐CO‐ of Boc), 166.96 (*C*_*s*_, 1,3‐COCH_2_ COOEt), 167.45 (*C*_*t*_, 1,3‐COCH_2_COOEt), 172.46 (*C*_*u*_, Lys C^*α*^‐COO^t^Bu).

## Conflict of Interest

Kousaku Ohkawa reports that financial support was provided by Japan Society for the Promotion of Science (JSPS). Ichiro Yuki reports that financial support was provided by National Institutes of Health. Kousaku Ohkawa, Parisa Khosropour, Shuichi Suzuki, Frank P. K. Hsu, and Ichiro Yuki report a relationship with AquaTex Medical, Inc that includes board membership, employment, and equity or stocks. Other authors declare that they have no known competing financial interests or personal relationships that could have appeared to influence the work reported in this paper.

## Author Contributions

**Kousaku Ohkawa**: conceptualization (lead); data curation (lead); formal analysis (lead); funding acquisition (lead); investigation (lead); methodology (lead); project administration (lead); resources (lead); software (lead); validation (lead); visualization (lead); writing—original draft (lead); writing—review and editing (lead). **Tracy Nguyen**: data curation (supporting); formal analysis (supporting); methodology (supporting); resources (supporting); software (supporting). **Chloe Jin**: data curation (supporting); formal analysis (supporting); methodology (supporting). **Hemdeep Kaur**: data curation (supporting); formal analysis (supporting); methodology (supporting). **Beatrice Mae Malvar**: data curation (supporting); formal analysis (supporting); methodology (supporting). **Rebecca Back**: data curation (supporting); formal analysis (supporting); methodology (supporting). **Parisa Khosropour**: project administration (supporting); resources (lead); supervision (lead). **Shuichi Suzuki**: funding acquisition (supporting); project administration (supporting); resources (lead); supervision (lead). **Frank P. K. Hsu**: funding acquisition (lead); resources (supporting); supervision (supporting). **Ichiro Yuki**: conceptualization (supporting); data curation (lead); funding acquisition (lead); resources (lead); supervision (lead).

## Supporting information

Supplementary Material

## Data Availability

The data that support the findings of this study are available in the supplementary material of this article.
