# Supplementary material for: Development of 5‐Amino‐2,4,6‐triiodoisophthalic Acid Derivatives for Carbamoylation of Amino Acids
Source: ChemistryOpen. 2025 Sep 12;14(12):e202500174. doi: 10.1002/open.202500174 (PMC12680579; doi:10.1002/open.202500174)
Supplement: Supplementary file 1 — Supplementary Material [file OPEN-14-e202500174-s001.pdf]

Supporting Information

Development of 5-amino-2,4,6-triiodoisophthalic acid derivatives for carbamoylation of amino acids

Kousaku Ohkawa[a,b,c,d*], Hemdeep Kaur[c], Tracy Nguyen[c], Chloe Jin[c], Beatrice Mae Malvar[c], Rebecca Christine Back[c], Parisa Khosropour[d], Shuichi Suzuki[c,d], Frank P. K. Hsu[c,d], Ichiro Yuki[c,d,e]

a Division of Synthetic Polymers, Institute of High Polymer Research, Faculty of Textile Science and Technology, Shinshu University, Tokida 3-15-1, Ueda 386-8567, Japan.

b Division of Bibliometrics and Social Implementation, Institute for Fiber Engineering, Interdisciplinary Cluster for Cutting Edge Research, Shinshu University, Tokida 3-15-1, Ueda 386-8567, Japan.

c Department of Neurological Surgery, University of California, Irvine, 200S Manchester St., Suite 210, Orange, CA92868, USA.

d AquaTeX Medical, Inc., Irvine, CA92617, USA

e Department of Neurosurgery, The Jikei Univerisity Hospital, 3 Chome-25-8 Nishi-Shinbashi, Minato City, Tokyo 105-8461, Japan

* To whom correspondence to be addressed.

E-mail address: kohkawa@shinshu-u.ac.jp (Kousaku Ohkawa)

Abbreviations and trivial names of compounds

ATIIPA:	5-amino-2,4,6-triiodoisophthalic acid
DEtTIIP:	diethyl 5-amino-2,4,6-triiodoisophthalate
DAcOEtTIIP:	di(acetoxyethyl) 5-amino-2,4,6-triiodoisophthalate
DEtTIIP-NCO:	diethyl 5-isocyanato-2,4,6-triiodoisophthalate
DAcOEtTIIP-NCO:	di(acetoxyethyl) 5-isocyanato-2,4,6-triiodoisophthalate
Iohexol:	5-[*N*-(2,3-dihydroxypropyl)acetamido]-2,4,6-triiodo-*N*,*N*'-bis(2,3-dihydroxypropyl) isophthalamide (isomer mixture)
Iopamidol:	*N*,*N*'-bis[2-hydroxy -1-(hydroxymethyl)ethyl]-5-[[(2*S*)-2-hydroxy -1-oxopropyl]amino] -2,4,6-triiodo-1,3-benzene dicarboxamide)
Iodixanol:	5,5'-[(2-hydroxypropane-1,3-diyl) bis(acetylazanediyl)]bis[N^1,N^3-bis(2,3-dihydroxypropyl)-2,4,6-triiodoisophthalamide]
Boc:	*tert*-butyloxycarbonyl
-OEt:	ethyl ester
-O^{t}Bu:	*tert*-butyl ester

ex.

DAcOEtTIIP:CO-*β*Ala-O^{t}Bu: di(acetoxyethyl) 5-(carbonyl-*β*-alanyloxy *tert*-butyl)amino-2,4,6-triiodoisophthalate

Synthesis and Identification of DEtTIIP and DEtTIIP-NCO

DEtTIIP: ATIIPA (5.00 g; 8.95 mmol; 1.00 eq.mol) was dissolved in absolute DMF (20 mL), and $NaHCO_3$ (3.16 g; 37.6 mmol; 4.2 eq.mol) was added to the solution. After evolution of CO_2 gas was stopped, bromoethane (4.18 mL; 5.85 g; 53.7 mmol; 6.00 eq.mol) was mixed with the suspension, and the reaction was continued for 18 hours at 45 °C. The reaction mixture was diluted with ethyl acetate (EtOAc, 180 mL), and the organic layer was washed with aqueous $NaHCO_3$ (5.0 (w/v)%) three times, with brine three times, subsequently dried on anhydrous Na_2SO_4. After evaporation of solvent under reduced pressure, the residue was crystallized with *n*-hexane and recovered by filtration. The crude product was recrystallized from EtOAc and *n*-hexane. Yield, 5.20 g (95.4%). DETIIP: (^{1}H-NMR, 400 MHz, DMSO*d6*): δ (ppm, TMS): 1.34 (t, 6H, J = 7.12 Hz, H_a, 1,3-COCH$_2$C$\underline{H_3}$), 4.34 (q, 4H, J = 7.11 Hz, H_b, 1,3-COC$\underline{H_2}$CH$_3$), 5.68 (s, 2H, H_c, 5-N$\underline{H_2}$); (^{13}C-NMR, 101 MHz, DMSO*d6*): δ (ppm, TMS): 14.28 (C_a, 1,3-CO-CH$_2$$\underline{C}H_3$), 62.58 ($C_b$, 1,3-CO-$\underline{C}H_2CH_3$), 71.74 ($C_c$, $\underline{C^2}$-I), 79.93 (C_d, $\underline{C^{4,6}}$-I), 146.93 (C_e, $\underline{C^{1,3}}$-CO-), 148.67 (C_f, $\underline{C^5}$-NH$_2$), 168.51 (C_g, 1,3-$\underline{C}$OOEt).

DEtTIIP-NCO: DEtTIIP (1.50 g; 2.44 mmol; 1.00 eq.mol) was dissolved in absolute 1,4-dioxane (10 mL). To this solution added were dried charcoal powder (0.05 g) and bis(trichloromethyl)carbonate (TCMC, 2.17 g; 3.00 eq.mol of $COCl_2$ *in situ*), and then the reaction was continued for 6 hours at 75 °C. After annealing of the reaction mixture, the charcoal powder was removed by filtration, and the solvent and excess $COCl_2$ was evaporated under reduced pressure. The residue was completely dried *in vacuo* for 2 hours at 55 °C. After confirmation of the NCO stretching vibration at 2264 cm^{-1} by the infrared spectroscopy, the oily product was immediately used for the carbamoylation reactions. Yield, 1.55 g (99.1 mol%).

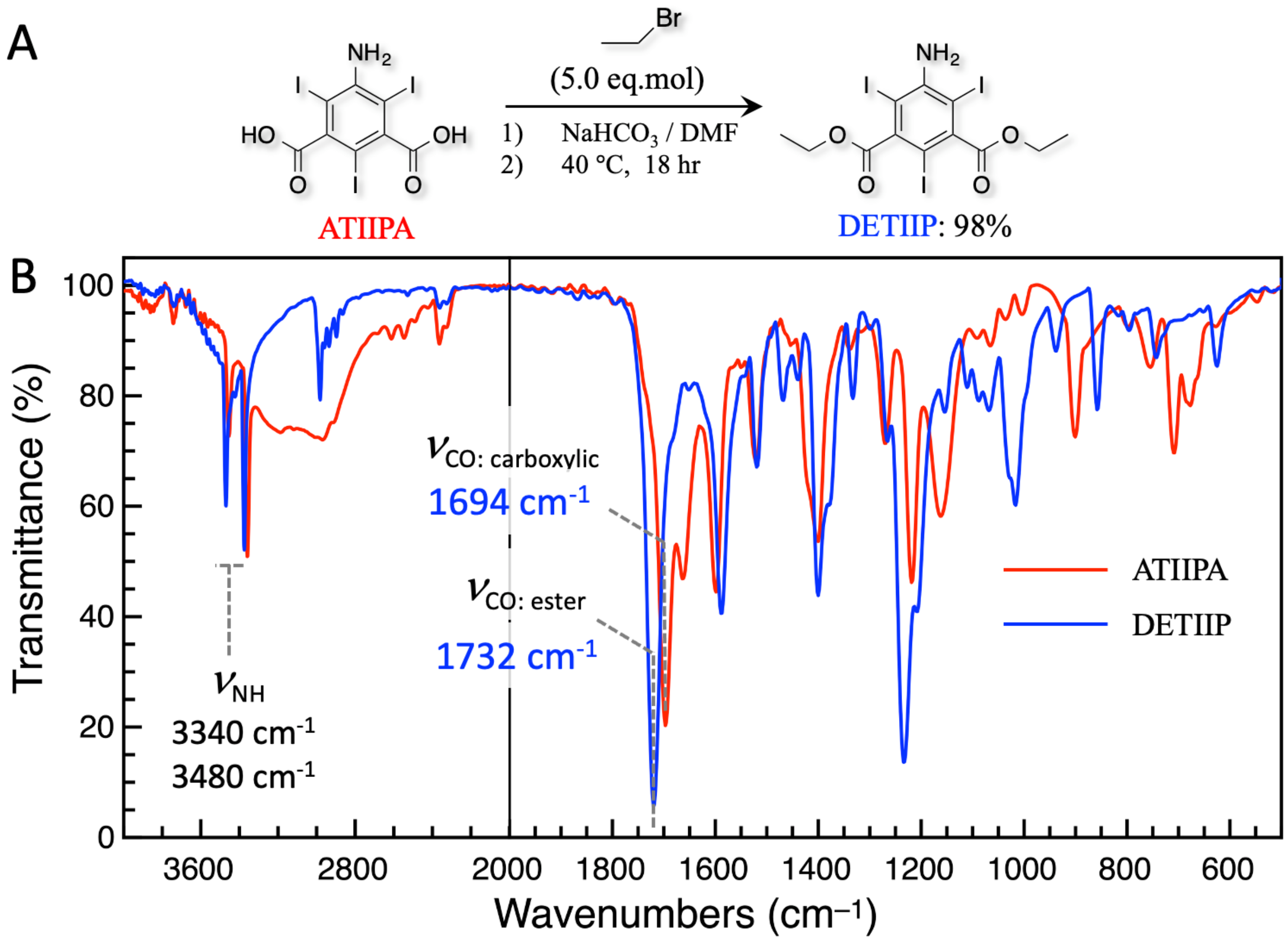

Figure S1. (A) base-catalyzed esterification of ATIIPA using ethyl α-bromoethane to produce DEtTIIP, and (B) infrared spectra of the substrate ATIIPA and the product DEtTIIP.

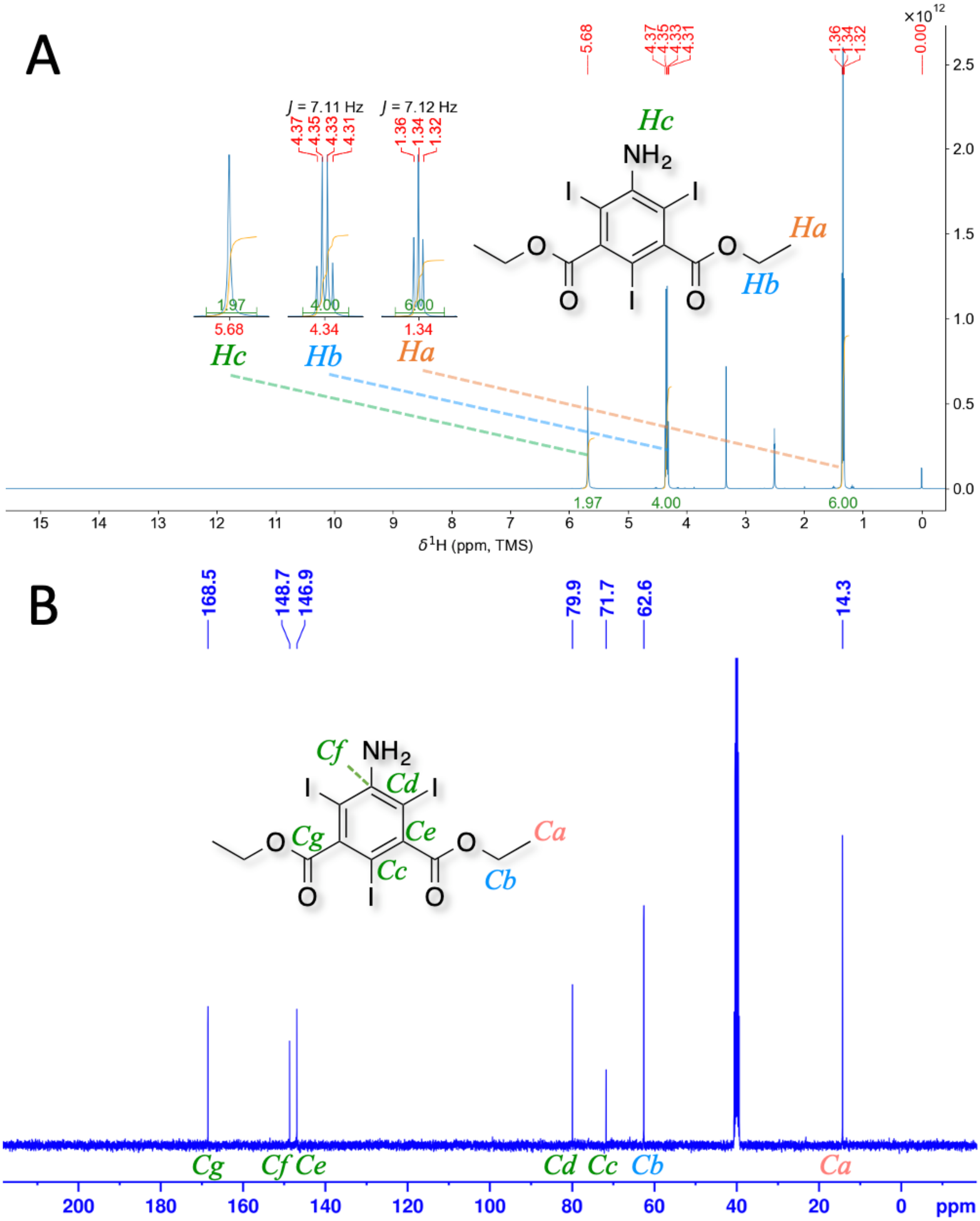

Figure S2. (A) ^{1}H- and (B) ^{13}C-CPD spectra of DEtTIIP.

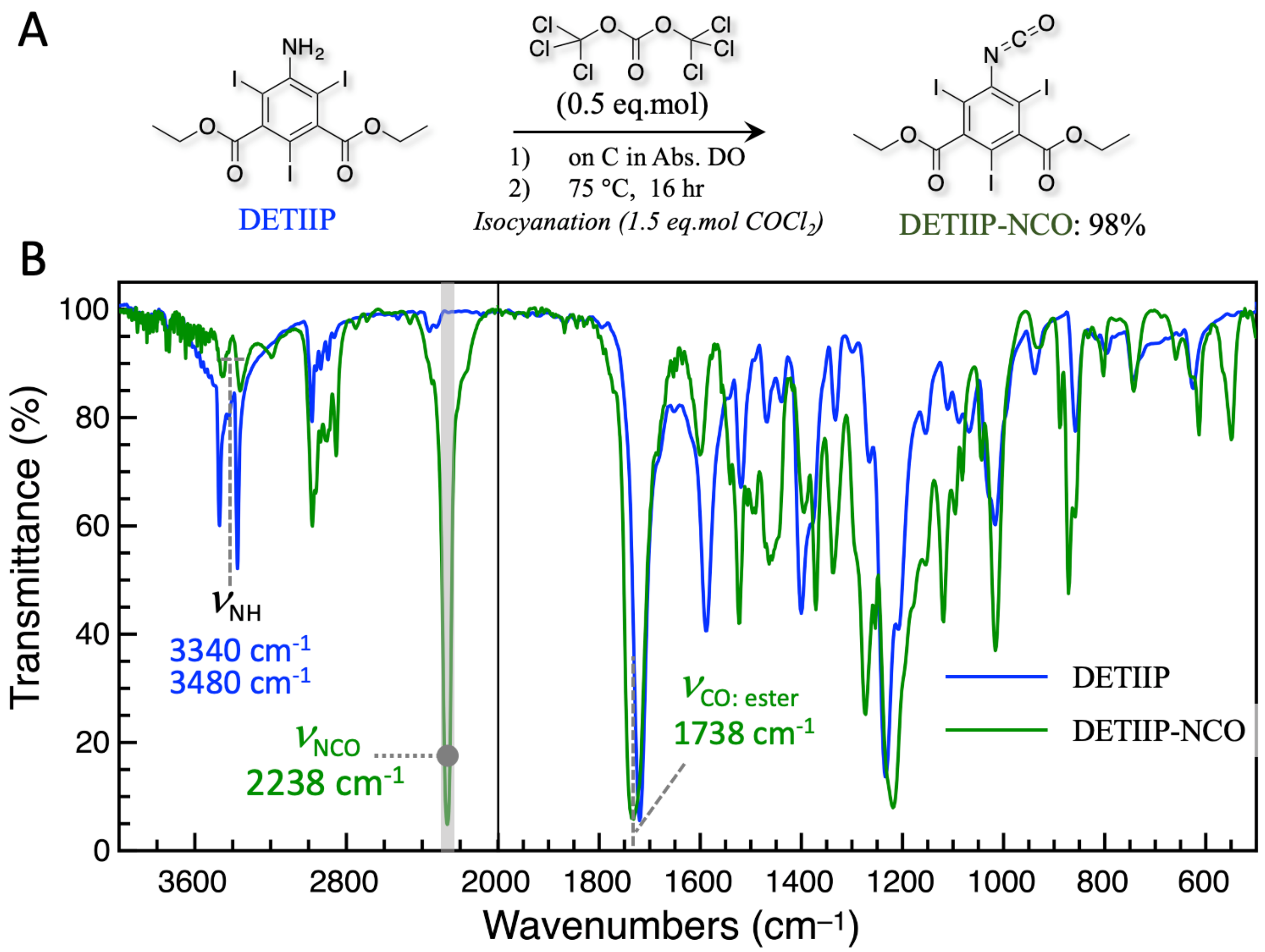

Figure S3. (A) conversion of 5-amino to 5-isocyanato groups of DEtTIIP to give DEtTIIP-NCO, and (B) infrared spectra of the substrate DEtTIIP and the product DEtTIIP-NCO.
